# Experimental and numerical investigation on the reinforced concrete boundary beam-wall system subjected to axial compression

**DOI:** 10.1371/journal.pone.0301622

**Published:** 2024-04-17

**Authors:** Dae-Jin Kim, Jin-Ju Han, Seung-Il Kim

**Affiliations:** 1 Department of Architectural Engineering, Kyung Hee University, Yongin, Gyeonggi, Republic of Korea; 2 Housing Construction Method Development Team, DL E&C, Ltd., Seoul, Republic of Korea; Jamia Millia Islamia, INDIA

## Abstract

This paper proposes a reinforced concrete (RC) boundary beam-wall system that requires less construction material and a smaller floor height compared to the conventional RC transfer girder system. The structural performance of this system subjected to axial compression was evaluated by performing a structural test on four specimens of 1/2 scale. In addition, three-dimensional nonlinear finite element analysis was also performed to verify the effectiveness of the boundary beam-wall system. Three test parameters such as the lower wall length-to-upper wall length ratio, lower wall thickness, and stirrup details of the lower wall were considered. The load-displacement curve was plotted for each specimen and its failure mode was identified. The test results showed that decrease in the lower wall length-to-upper wall length ratio significantly reduced the peak strength of the boundary beam-wall system and difference in upper and lower wall thicknesses resulted in lateral bending caused by eccentricity in the out-of-plane direction. Additionally, incorporating cross-ties and reducing stirrup spacing in the lower wall significantly improved initial stiffness and peak strength, effectively minimizing stress concentration.

## 1. Introduction

In recent years, in South Korea, the construction of underground parking facilities or commercial spaces in the lower levels has become a common practice for efficient land usage in apartment buildings [1, 2]. As a result, one of the most widely used structural systems in the apartment buildings is the reinforced concrete (RC) upper-wall and lower-frame structural system. This system features wall structures on the upper floors for residential spaces, while the lower floors are designed with beam-column frames to accommodate parking and commercial areas.

**Fig 1:**
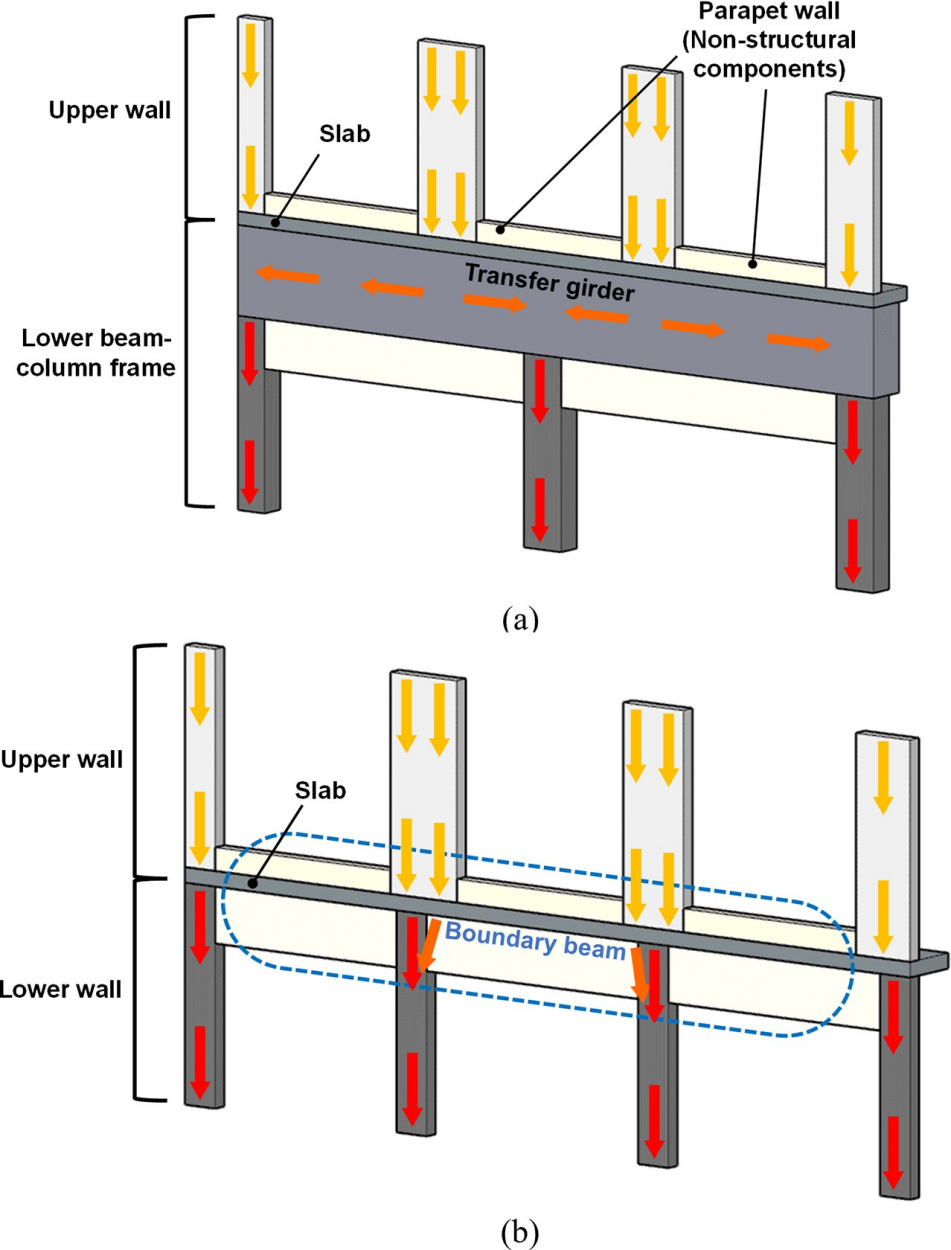
Comparison between the boundary beam and conventional transfer girder systems. (a) Conventional transfer girder system. (b) Newly proposed boundary beam-wall system.

To efficiently distribute loads from the upper walls to the lower frames in apartment buildings, large transfer girders are often employed [3, 4]. For buildings with fewer than 20 floors, these girders typically have a depth of around 2 meters, while for taller buildings over 20 floors, the depth increases to about 2.7 meters [5]. However, the substantial size of these girders requires extensive use of concrete and reinforcing steel bars, complicating construction processes like concrete pouring and formwork. This can lead to construction delays and increased costs. Furthermore, the conventional transfer girder system, as shown in Fig 1(A), transfers vertical loads from the upper walls to lower columns in an indirect and unstable manner. This often results in uneven load distribution and localized stress concentration, posing structural challenges [6–9].

In the conventional RC transfer girder system, a soft story may form in the lower frame due to a vertical discontinuity in lateral stiffness and strength. This structural vulnerability can lead to excessive damage under lateral loads such as wind and earthquakes. To understand this effect, researchers have evaluated the seismic performance of the system using methods like pseudo-dynamic tests [10] and nonlinear finite element analysis [11]. These studies have shown that damage tends to be excessively concentrated in the lower frame columns. To prevent soft-story failure, various energy-dissipating devices have been explored, including viscous dampers [12], damping walls [13] and isolation systems designed to limit structural deformation [14].

**Fig 2:**
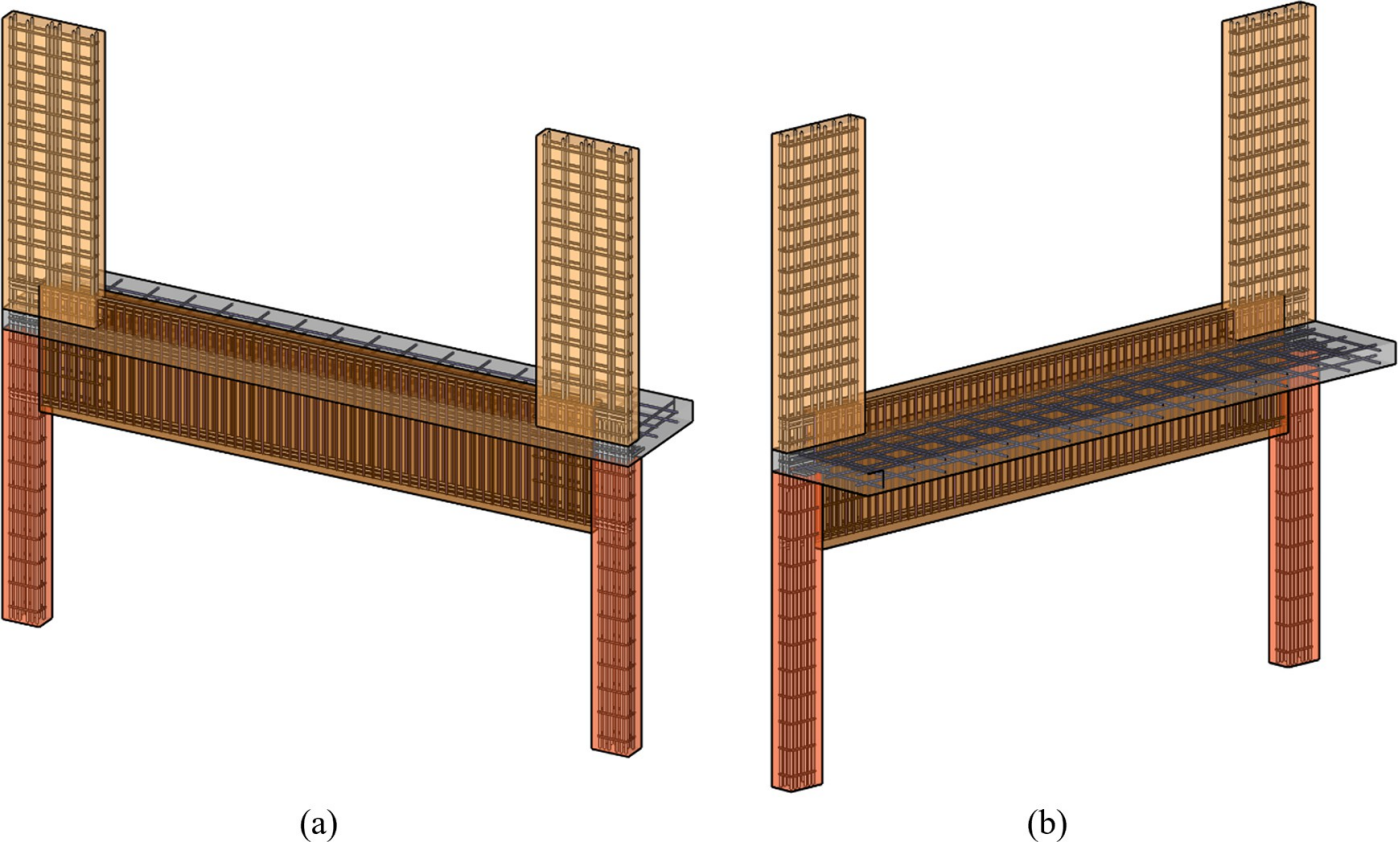
Typical shape and reinforcement details of the boundary beam-wall system. (a) Front view. (b) Back view.

To overcome the identified shortcomings in the conventional RC transfer girder system, this study suggests a novel RC boundary beam-wall system. In the proposed system, as illustrated in Fig 1(B), the lower columns are replaced with walls of reduced width compared to the upper ones. These lower walls are continuously aligned under the upper ones, enabling a relatively continuous vertical load transfer. Additionally, the transfer girder is replaced by a scaled-down boundary beam, a type of perimeter beam. Furthermore, the parapet walls with minimal reinforcement, traditionally considered non-structural elements in the design of conventional transfer girder systems, are integrated into the boundary beam and effectively contribute to the transfer of loads between the upper and lower wall components. This approach can significantly reduce the floor height where the transfer girders are installed, as well as the overall construction cost, without significantly compromising the overall structural performance. Fig 2 illustrates the typical shape and reinforcement details of this new system.

In the newly proposed RC boundary beam-wall system, seismic loads are primarily resisted by other lateral force-resistant components, such as core RC shear walls, while its primary structural components are designed to withstand vertical loads induced by gravity. Consequently, as the first study to investigate the structural performance of the new system, this research aims to evaluate its compressive performance through both experimental and analytical approaches. A structural test was conducted on four 1/2-scale specimens subjected to axial compression. Additionally, three-dimensional nonlinear finite element analysis was performed to validate the effectiveness of the boundary beam-wall system. The design requirements for the newly proposed boundary beam-wall system are also discussed.

This paper is outlined as follows. After the introduction, Section 2 presents the details of the experimental program, including information about the test specimens, testing equipment, and test procedure. In Section 3, the failure modes of the test specimens are identified and their load-displacement curves are analyzed. In addition, the detailed test results including strain distributions and load path identification are discussed. Section 4 encompasses the nonlinear finite element analysis of the boundary beam-wall system, followed by a comparison and analysis of the results in relation to the test data. Finally, Section 5 provides a summary and concluding remarks.

## 2. Experimental program

### 2.1. Test specimens

In this study, axial compression tests were conducted on a total of four specimens to evaluate the structural performance of the newly proposed boundary beam-wall system under axial compression loads. The test specimens were designed as two-story structures to investigate their structural behavior when axial loads are transferred from the upper wall to the lower wall. The dimensions of the test specimens were scaled down by half to accommodate the capacity of the testing equipment. The horizontal length of the upper wall was set at 1.0 meter (scaled-down dimension = 500 mm), and the thickness of the upper wall was 200 mm (scaled-down dimension = 100 mm), taking into consideration the typical window sizes found in apartment buildings in South Korea. The detailed reinforcement of the specimens was determined by referencing the design of an actual 28-story RC apartment building constructed in South Korea.

In the design of the four test specimens, three parameters such as the ratio of the lower wall length to the upper one, the thickness of the lower wall, and the details of the stirrup reinforcement in the lower wall were taken into consideration. The ratio of the lower wall length to the upper wall length is a parameter associated with the utilization of the lower-level space in the boundary beam-wall system. Since the lower level is typically used for parking spaces, reducing the length of the lower wall allows for accommodating larger-sized vehicles within the given space. However, this reduction in lower wall length may significantly affect the compressive strength of the lower walls, necessitating a design approach that considers both the utilization of the lower-level space and its impact on structural performance.

The thickness of the lower wall is a parameter related to meeting the minimum thickness requirements outlined in the practical design method of Korean Design Standard [15] for estimating the axial capacity of RC wall panels. According to this approach, the thickness of the wall shall be at least 1/25 of the lesser dimension between the unsupported length and unsupported height for bearing-type walls. Consequently, in order to satisfy this requirement, the thickness of the lower wall may often exceed that of the upper wall. This discrepancy can complicate the reinforcement detailing and formwork installation. Therefore, this study explores the structural implications of having equal thicknesses for the upper and lower walls, without adhering to the requirements of the practical design method.

According to the Korean Design Standard [15], RC wall panels shall be subjected to an axial force that is less than 0.4*A*_*g*_*f*_*ck*_. Additionally, the vertical reinforcement ratio in these panels shall not exceed 1%. If these conditions are not met, the structure must be designed as an RC column. In this study, the lower walls of the test specimens fall under the definition of RC columns, rather than that of RC wall panels. However, for general application, the boundary beam-wall system must also meet the requirements for RC wall panels as specified in the Korean Design Standard.

The stirrup reinforcement detail in the lower wall is crucial for its confinement effect, especially under axial compression in the boundary beam-wall system. As evidenced in the test results from Section 3.1 and the finite element analysis in Section 4.2, stress concentration tends to occur near the top of the lower wall. Generally, closer stirrup spacing can alleviate this stress concentration by improving the confinement effect of concrete. Consequently, this study investigates the impact of using stirrup reinforcement with reduced spacing, along with the addition of cross-ties, on the overall performance of the boundary beam-wall system.

**Fig 3:**
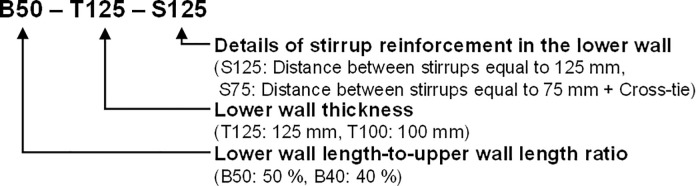
Specimen identification.

**Fig 4:**
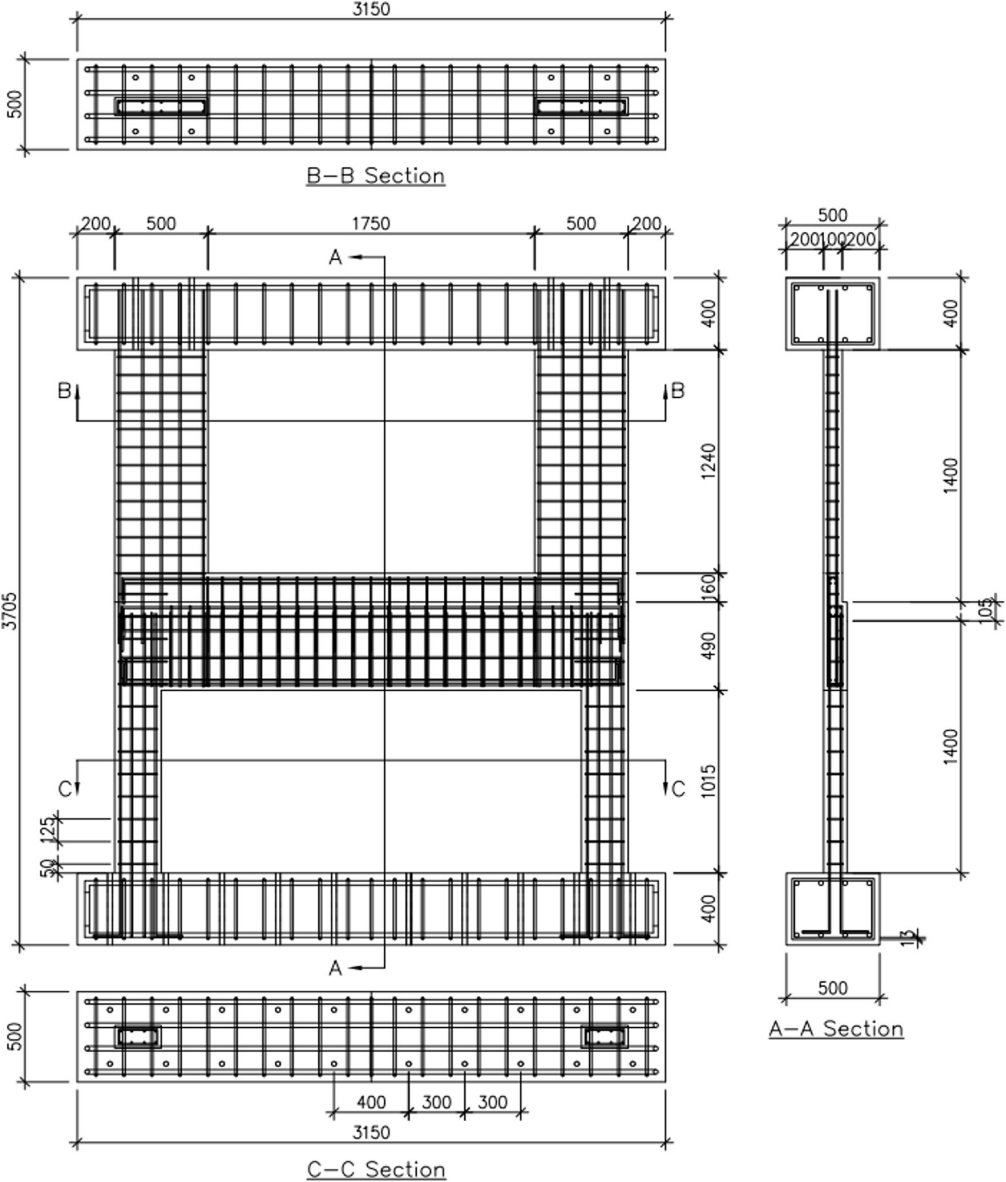
Details of the reference specimen (B50-T125-S125). (Unit: mm).

**Fig 5:**
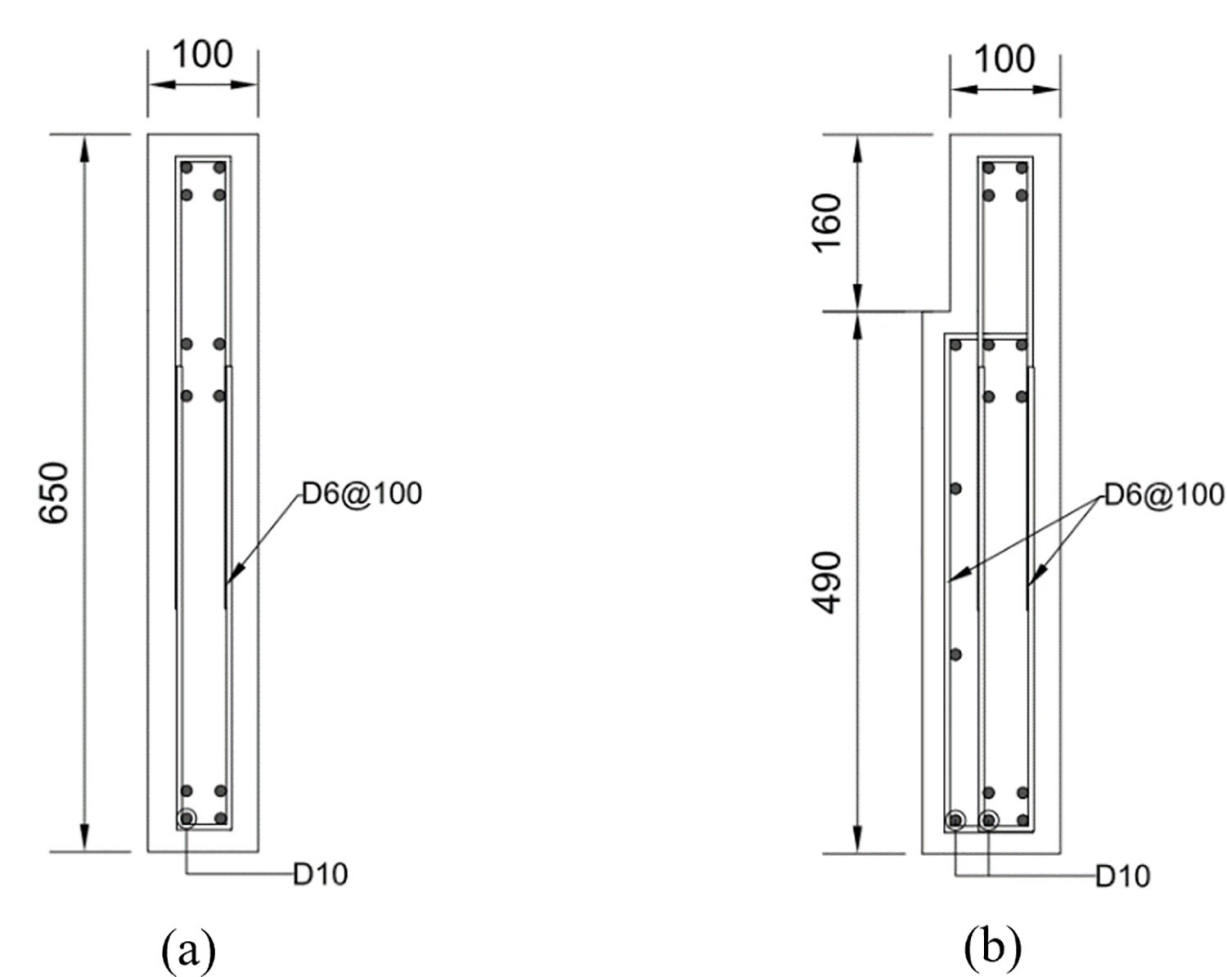
Boundary beam section details of the test specimens. (Unit: mm). (a) B50-T100-S125. (b) The other three specimens.

**Fig 6:**
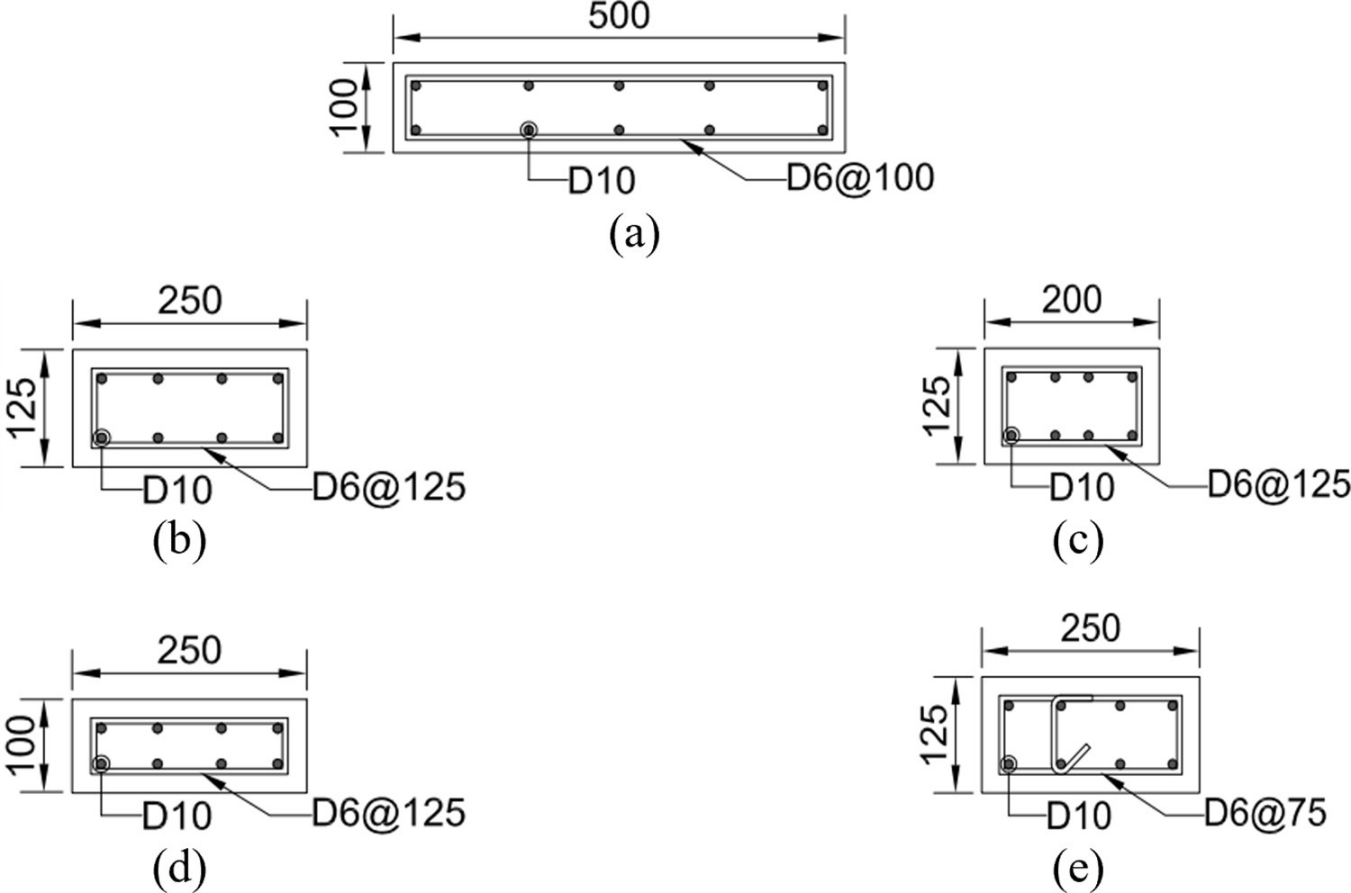
Wall section details of the test specimens. (Unit: mm). (a) Upper wall. (b) Lower wall of specimen B50-T125-S125. (c) Lower wall of specimen B40-T125-S125. (d) Lower wall of specimen B50-T100-S125. (e) Lower wall of specimen B50-T125-S75.

The four test specimens, detailed in Table 1 and identified in Fig 3, were designed considering various factors. Fig 4 provides the dimensions and reinforcement details of the reference specimen, B50-T125-S125. While all specimens share identical upper wall dimensions and reinforcement, they differ in lower wall features based on specific test parameters, as indicated in Figs 5 and 6. Specimen B40-T125-S125 explores the effects of reducing the lower to upper wall length ratio to 40% as illustrated in Fig 6(C), while keeping the lower wall thickness and stirrup spacing consistent with the reference. Specimen B50-T100-S125, with both walls having a uniform thickness of 100 mm as illustrated in Fig 5(A), examines the consequences of deviating from the practical design method’s standards. Finally, specimen B50-T125-S75, as shown in Fig 6(E), is similar to the reference, but with a decreased stirrup spacing of 75 mm and the addition of cross-ties in the lower wall’s stirrup reinforcement. The dimensions presented in the table and figures are scaled values using the 1/2 reduction factor previously mentioned.

**Table 1:** Summary of test specimens.

Specimen	Lower wall length-to-upper wall length ratio (%)	Lower wall thickness (mm)	Distance between stirrups (mm)	Existence of cross-ties
B50-T125-S125	50	125	125	No
B40-T125-S125	40			
B50-T100-S125	50	100		
B50-T125-S75		125	75	Yes

Normal strength concrete was used in the manufacturing of the test specimens. The compressive strength of the concrete was measured in accordance with ASTM C39 [16], and the test results are presented in Table 2, along with the concrete mix proportions. The compressive strength value in the table represents the average obtained from the results of six cylindrical concrete specimens. D10 rebars were used for both the upper and lower walls as well as the boundary beams, while D6 rebars were used for stirrup reinforcement of the lower wall. The yield and ultimate tensile strengths of these rebars were measured following the ASTM A370 standard [17] and are summarized in Table 3. For both D10 and D6 rebars, three specimens were tested. The average yield strengths were found to be 481 MPa for D10 and 317 MPa for D6, respectively. The values of coefficients of variation (COV) are also included in Tables 2 and 3.

**Table 2:** Concrete material properties.

Design strength (MPa)	W/C (%)	S/a (%)	Unit weight (kg/m^3^)					Compressive strength	
			W	C	Fine aggregate	Coarse aggregate	Air entraining admixture	Average (MPa)	COV (%)
30	41.8	45.8	115	395	812	981	2.8	31.7	3.54

**Table 3:** Rebar material properties.

Rebar	Yield strength		Ultimate strength		Modulus of elasticity	
	Average (MPa)	COV (%)	Average (MPa)	COV (%)	Average (MPa)	COV (%)
D10	481	0.68	565	0.94	179,719	2.71
D6	317	1.81	347	2.32	176,660	3.08

### 2.2. Testing equipment and procedure

**Fig 7:**
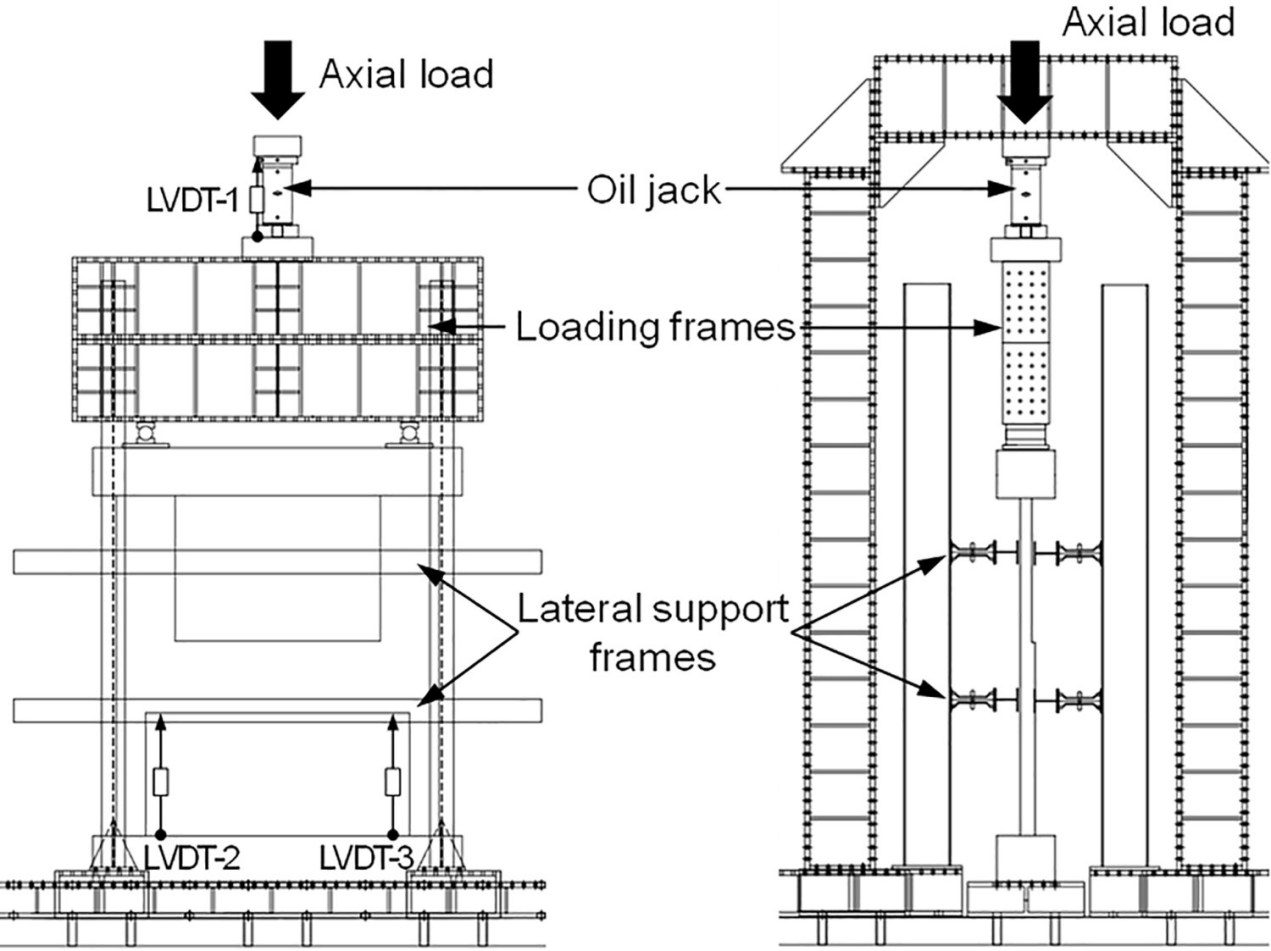
Test setup.

The compressive performance of the boundary beam-wall system was evaluated through a two-point loading test conducted on the four specimens discussed earlier. A vertical axial load was applied using an oil jack with a maximum capacity of 300 tons at a rate of approximately 1 kN/sec. To prevent bearing failure at the loading point, a steel plate with a size of 400mm × 400mm × 35mm was installed. Additionally, lateral support frames were set up on both sides of the test specimen to prevent any possible excessive deformation in the out-of-plane direction. This experimental setup is illustrated in Fig 7.

**Fig 8:**
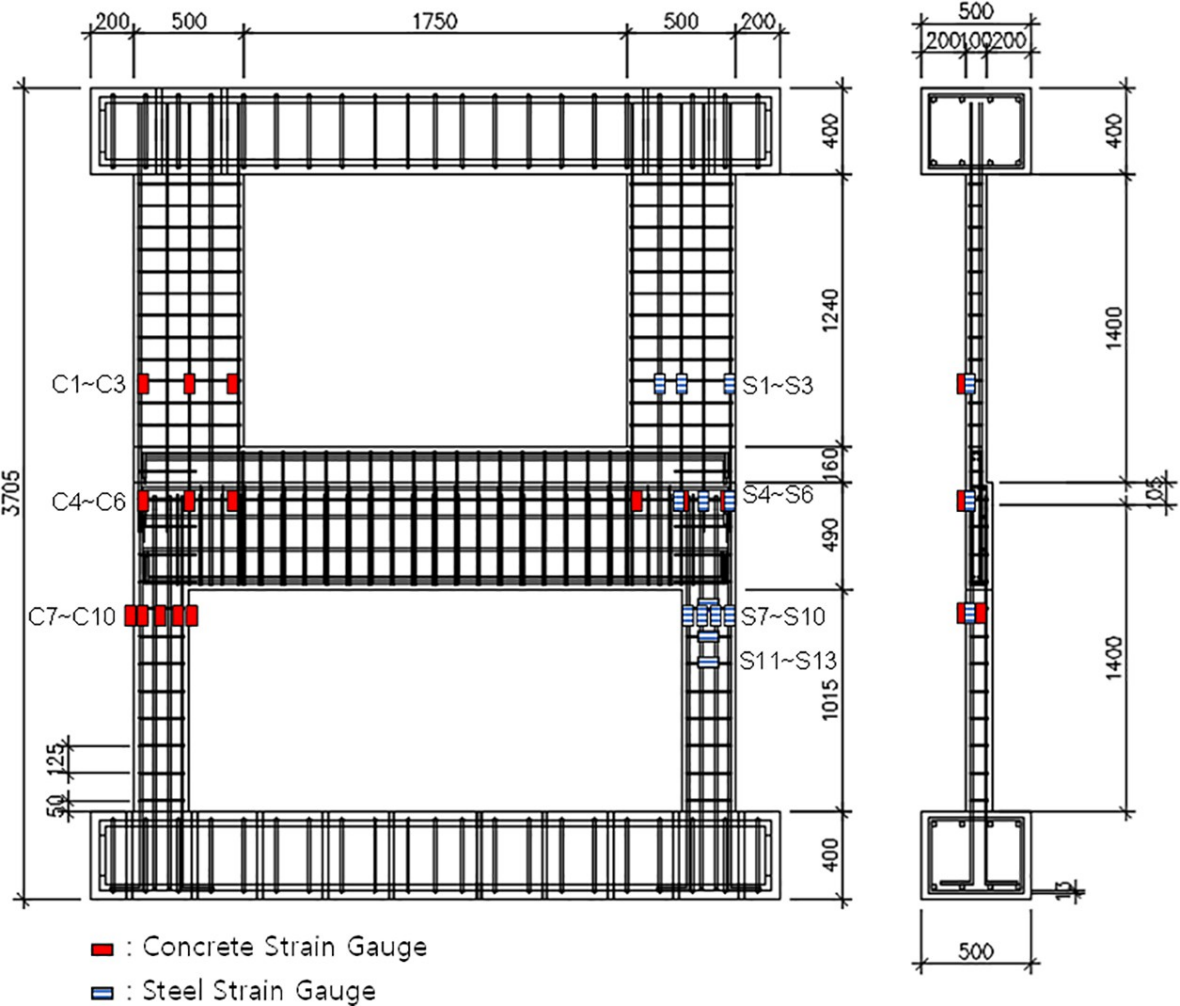
Strain gauge setup.

For each specimen, a total of three LVDTs (linear vertical displacement transducers) were utilized to measure displacement, as illustrated in Fig 7. To monitor the overall displacement of the specimen, one LVDT was installed at the position of the oil jack. Additionally, two LVDTs were positioned beneath the boundary beam to measure its vertical displacements. To trace the load transfer path, standard single wire strain gauges were applied to the concrete walls at three vertically different locations, namely C1~C3, C4~C6 and C7~C10, utilizing a polyester bonding adhesive. These strain gauges featured a gauge length of 60 mm and a strain limit of 2%. Additionally, strain gauges were affixed to the main rebars of the walls, denoted as S1~S3, S4~S6, and S7~S10. In order to investigate the effect of stirrup reinforcement details, three additional strain gauges (S11~S13) were attached to the stirrup near the top of the lower wall in both the reference specimen and specimen B50-T125-S75. The strain gauges for rebars had a length of 3 mm with a strain limit of 2%. The attachment positions of the strain gauges are indicated in Fig 8.

## 3. Test results and analysis

In this section, the failure modes of the test specimens are identified and their load-displacement curves are analyzed. In addition, the detailed test results such as strain distributions and load path identification are discussed.

### 3.1. Failure modes and load-displacement curves

**Fig 9:**
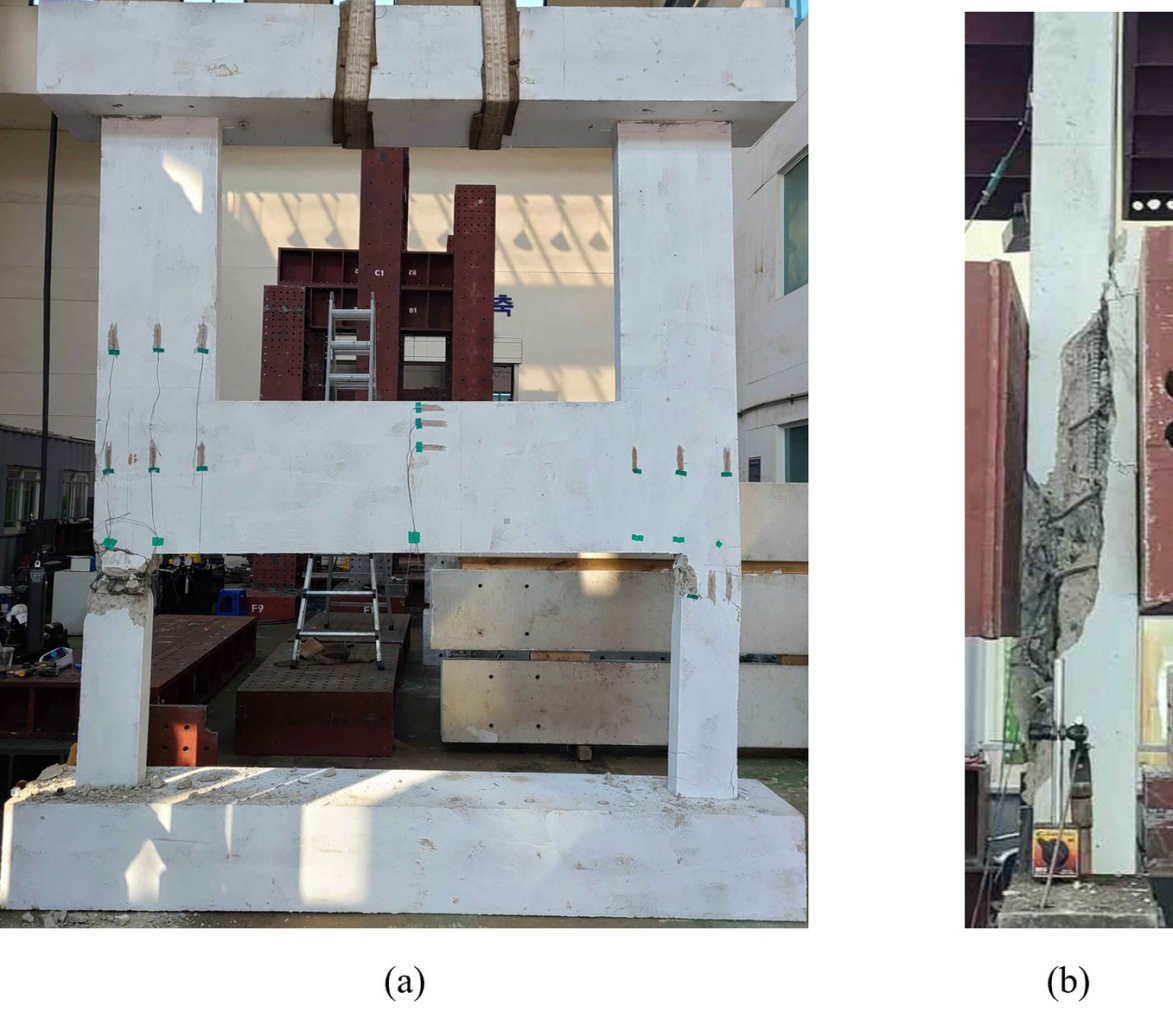
Typical failure shape (Reference specimen B50-T125-S125). (a) Front view. (b) Side view.

The typical failure shape and crack patterns of the test specimens are shown in Fig 9, which is the result of the reference specimen (B50-T125-S125). All test specimens did not exhibit significant cracks or deformation until the applied vertical load reached its peak value. At the point of peak load, concrete crushing occurred on the upper inner side of the lower wall, and concurrently, the main bars of the lower wall buckled, eventually resulting in a rapid and catastrophic failure, as shown in Fig 9(B).

**Fig 10:**
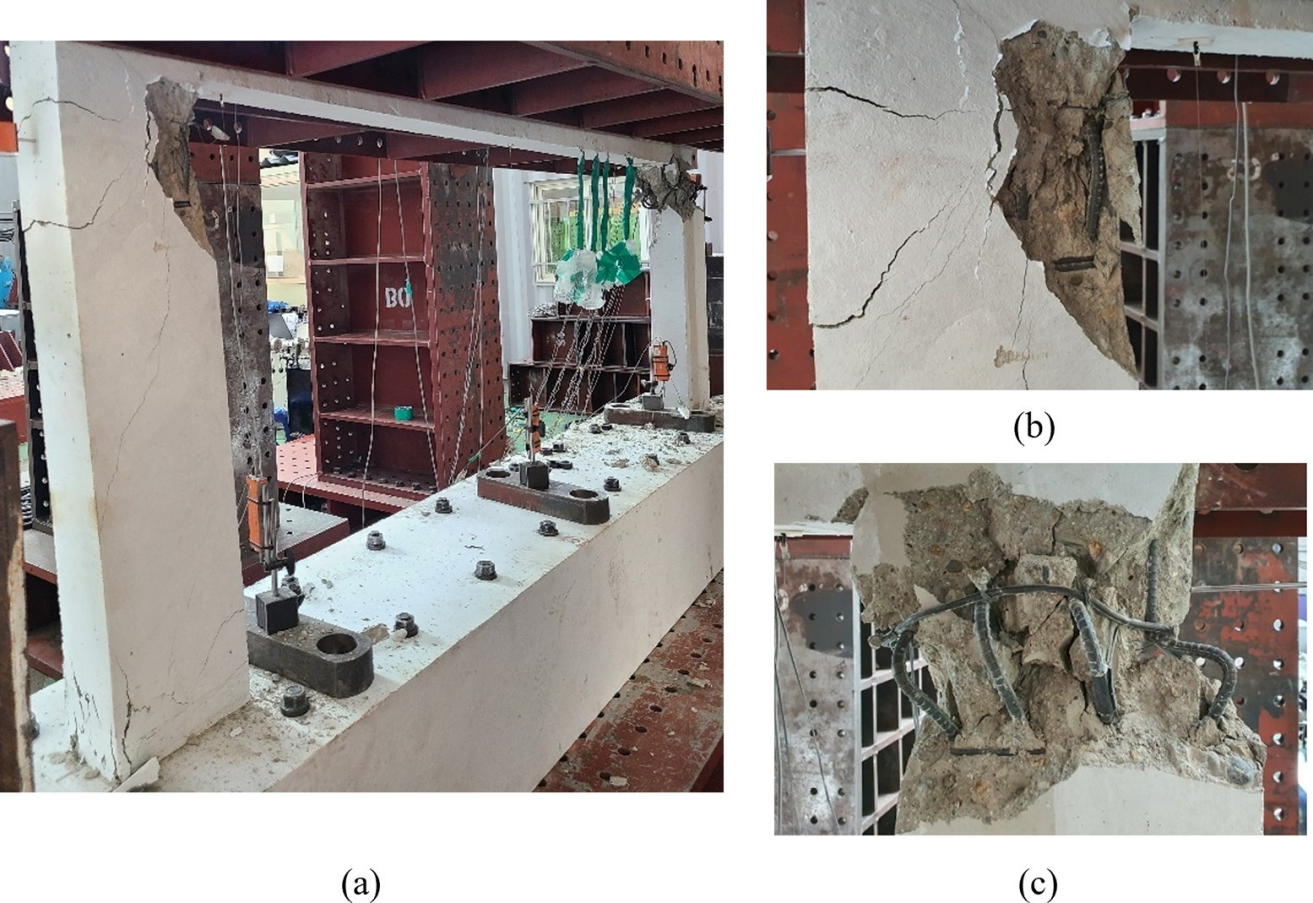
Lower wall failure shape of specimen B50-T125-S125. (a) Overall view. (b) Left lower wall. (c) Right lower wall.

**Fig 11:**
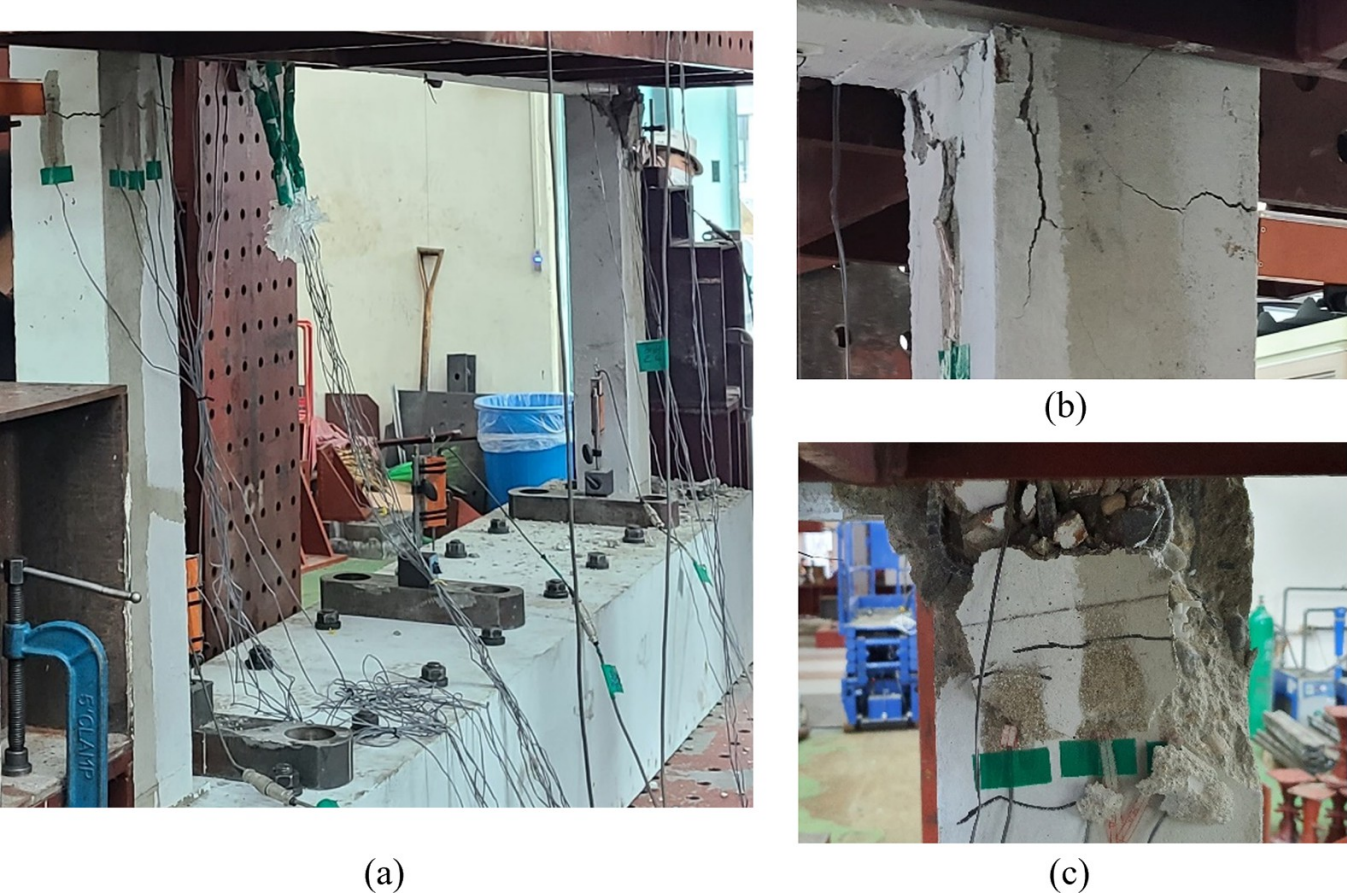
Lower wall failure shape of specimen B40-T125-S125. (a) Overall view. (b) Left lower wall. (c) Right lower wall.

**Fig 12:**
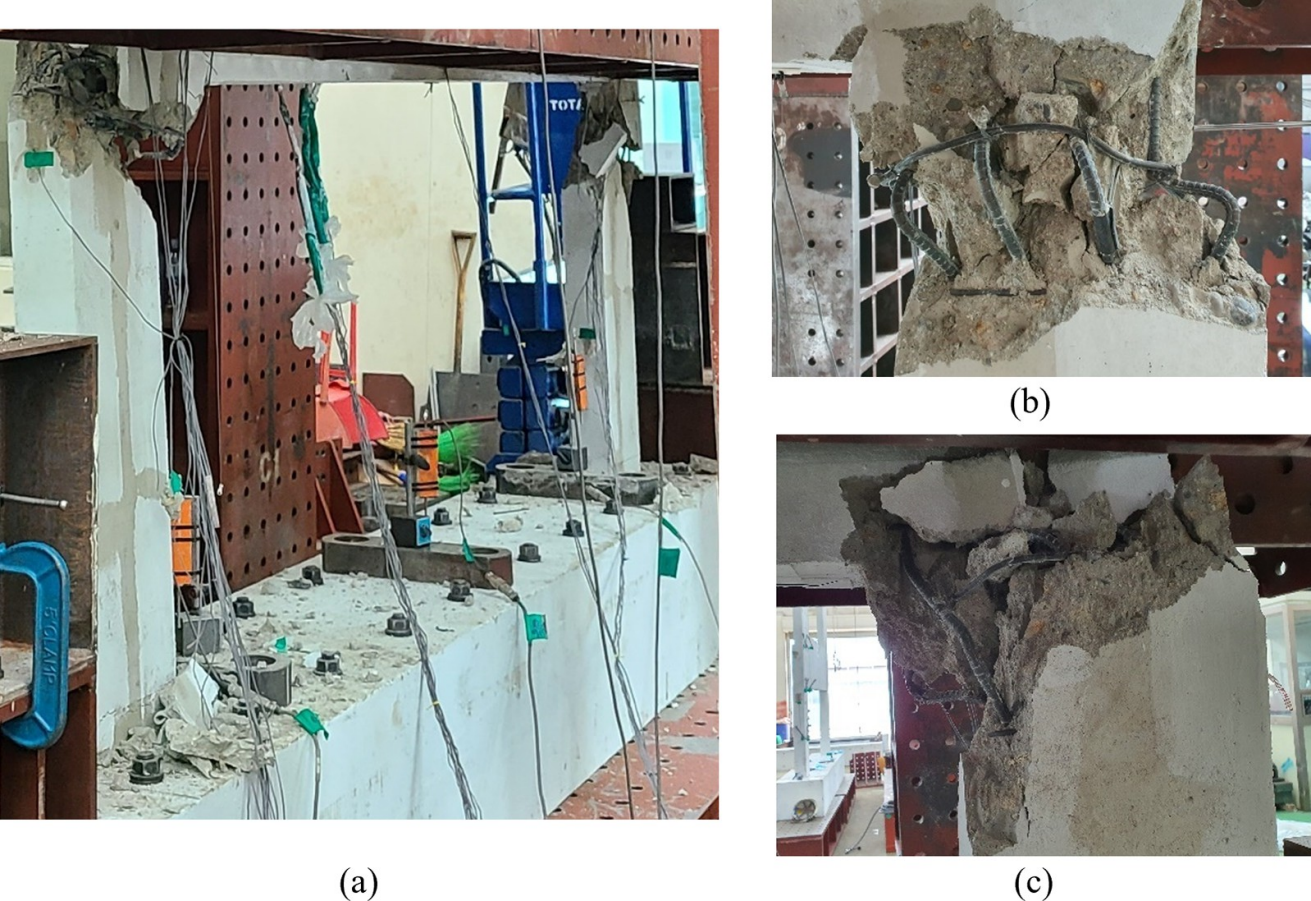
Lower wall failure shape of specimen B50-T100-S125. (a) Overall view. (b) Left lower wall. (c) Right lower wall.

**Fig 13:**
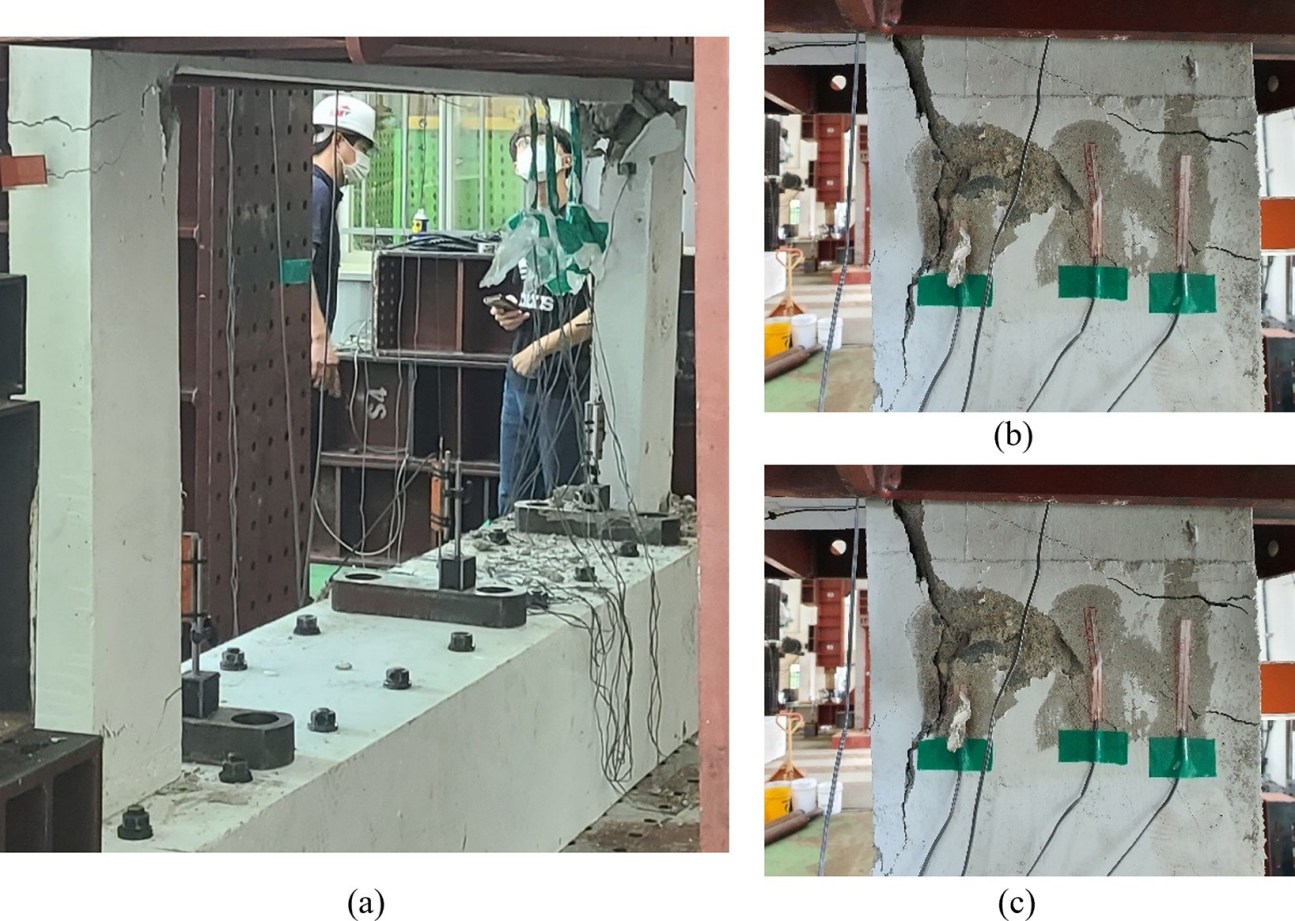
Lower failure shape of specimen B50-T125-S75. (a) Overall view. (b) Left lower wall. (c) Right lower wall.

Figs 10 to 13 show the final failure modes of the four test specimens, respectively. In all test specimens, both of the lower walls were severely damaged, with the relatively weaker one experiencing more significant damage. Additionally, it is noticeable that the lower walls exhibited slight outward bending. This can be attributed to the application of a vertical load with respect to the centerline of the upper wall, inducing bending moments in the lower walls due to its eccentricity. No cracks were observed in the upper wall or the boundary beams, except for some minor flexural cracks resulting from the damage that occurred during the delivery of the test specimens.

**Fig 14:**
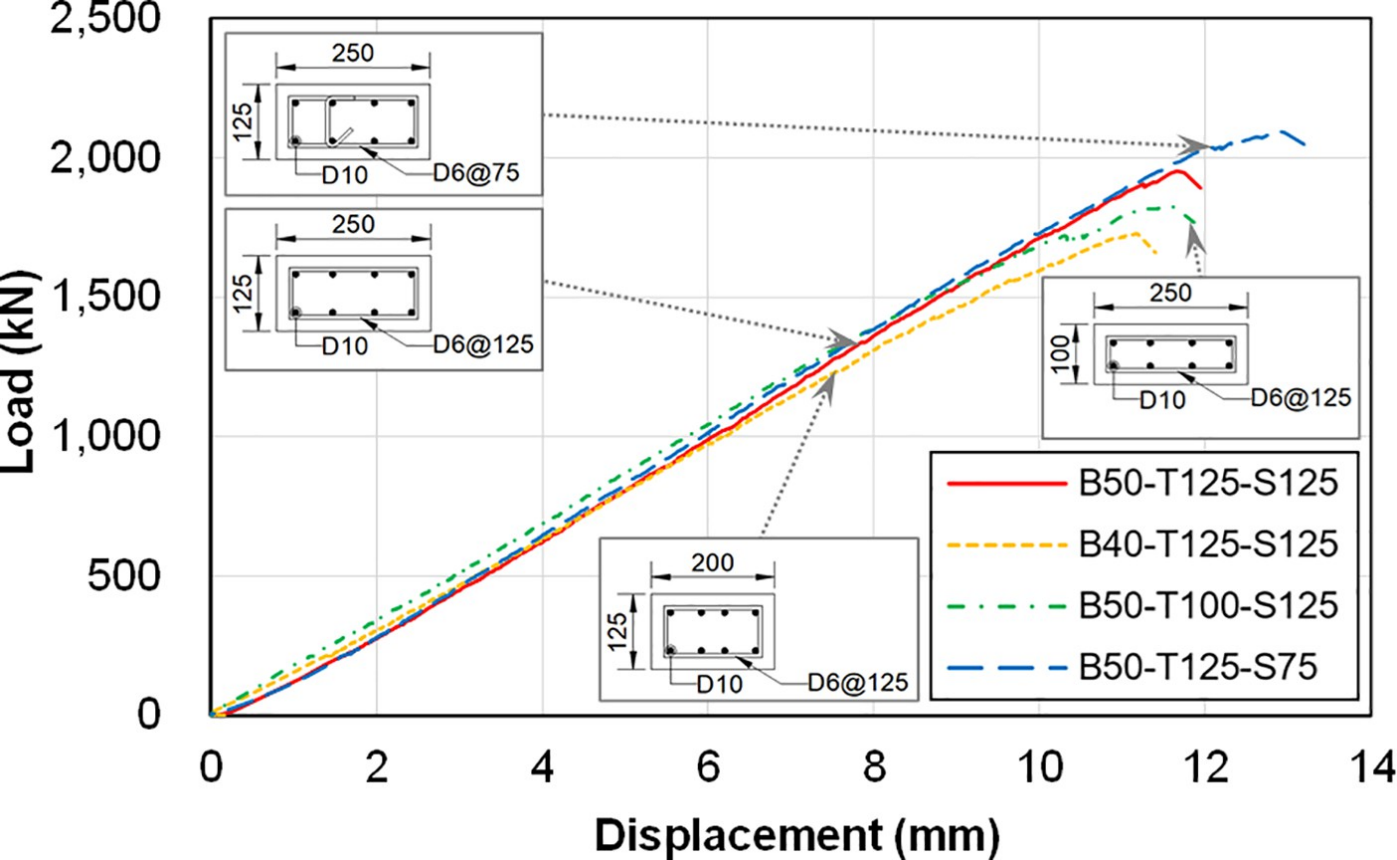
Load-displacement curves of the test specimens (LVDT-1).

Fig 14 plots the load-deflection curves for all four test specimens, with displacement measurements taken at the oil jack (LVDT-1). Table 4 summarizes the test results, including initial stiffness, peak strength and displacement at peak strength. It also provides the relative values of the initial stiffness and peak strength, which are estimated with respect to the reference specimen (B50-T125-S125). The initial stiffness is defined as the slope of the line connecting the origin to a point that represents 40% of the peak load.

**Table 4:** Test results.

Specimen	Displacement at peak strength (mm)	Initial stiffness (kN/mm)	Relative initial stiffness (%)	Peak strength (kN)	Relative peak strength (%)
B50-T125-S125	11.66	160.9	100.0	1,951.36	100.0
B40-T125-S125	11.18	159.0	98.8	1,724.75	88.4
B50-T100-S125	11.54	171.6	106.7	1,823.61	93.5
B50-T125-S75	12.90	166.1	103.2	2,092.81	107.2

The results of the table reveal that specimen B40-T125-S125 experienced a 1.2% reduction in initial stiffness and an 11.6% decrease in peak strength compared to the reference specimen. This suggests a significant impact on peak strength due to the decrease in the lower wall length-to-upper wall length ratio. In contrast, specimen B50-T100-S125 exhibited a 6.6% increase in initial stiffness but a 6.5% reduction in peak strength compared to the reference specimen. This is intriguing since both specimens, B40-T125-S125 and B50-T100-S125, share the same cross-sectional area and reinforcement detail for the lower wall. The distinct result can be attributed to the fact that specimen B50-T100-S125 had identical upper and lower wall thicknesses. This resulted in higher initial stiffness and peak strength, as it avoided lateral bending caused by eccentricity in the out-of-plane direction.

Lastly, specimen B50-T125-S75 demonstrated a 3.2% increase in initial stiffness and a 7.2% increase in peak load compared to the reference specimen. This confirms that incorporating cross-ties and a smaller stirrup distance in the lower wall can enhance the initial stiffness and peak strength of the boundary beam-wall system, ultimately reducing stress concentration. Further discussion on this issue can be found in Section 3.2.

**Fig 15:**
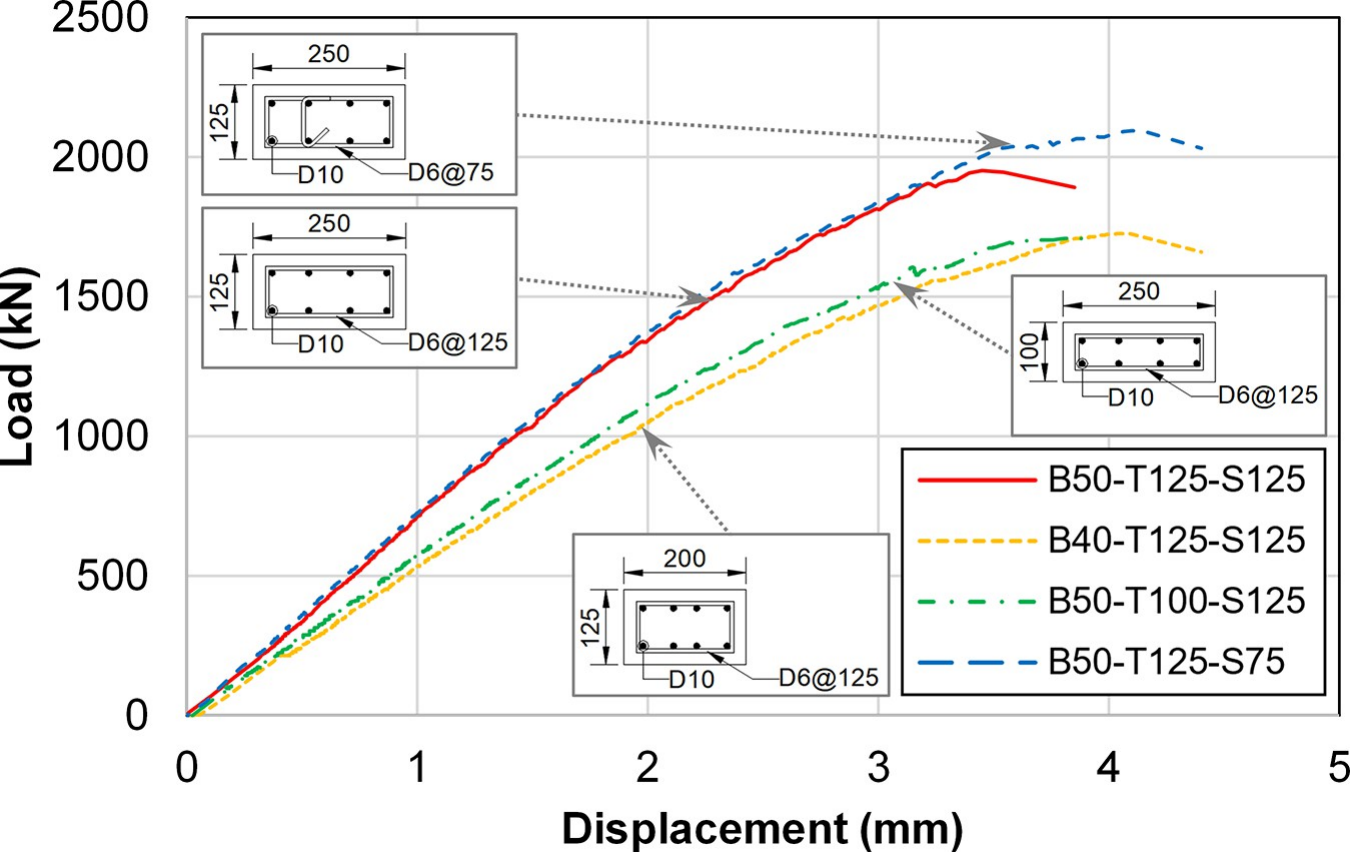
Load-displacement curves of the test specimens (LVDT-2, LVDT-3).

Fig 15 shows the load-displacement graph for the vertical displacement of the lower walls, with displacement measurements taken underneath both ends of the boundary beam (LVDT-2 and 3). The displacement values measured at LVDT-2 and 3 locations were nearly identical, and their average was utilized for analysis. Generally, the displacements shown in this figure are smaller than those in the previous one. The difference in the slopes of the curves is more pronounced because the locations of LVDT-2 and 3 are very close to the lower walls. These walls vary significantly in detail from one test specimen to another. Consequently, the results in Fig 15 inficate a more localized behavior than those in Fig 14.

### 3.2. Strain distribution and load path

**Fig 16:**
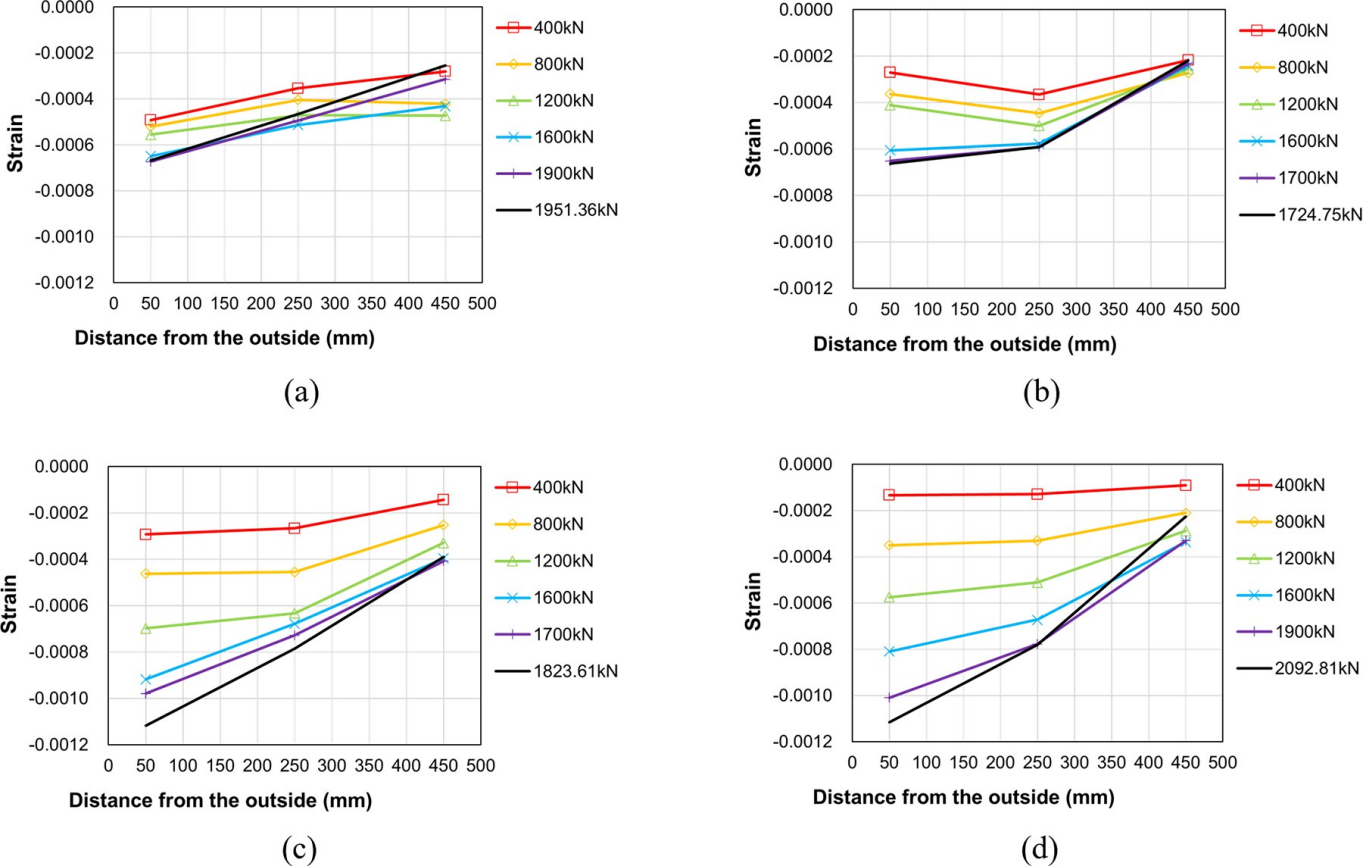
Concrete strain distribution at the upper wall (C1~C3). (a) Specimen B50-T125-S125. (b) Specimen B40-T125-S125. (c) Specimen B50-T100-S125. (d) Specimen B50-T125-S75.

**Fig 17:**
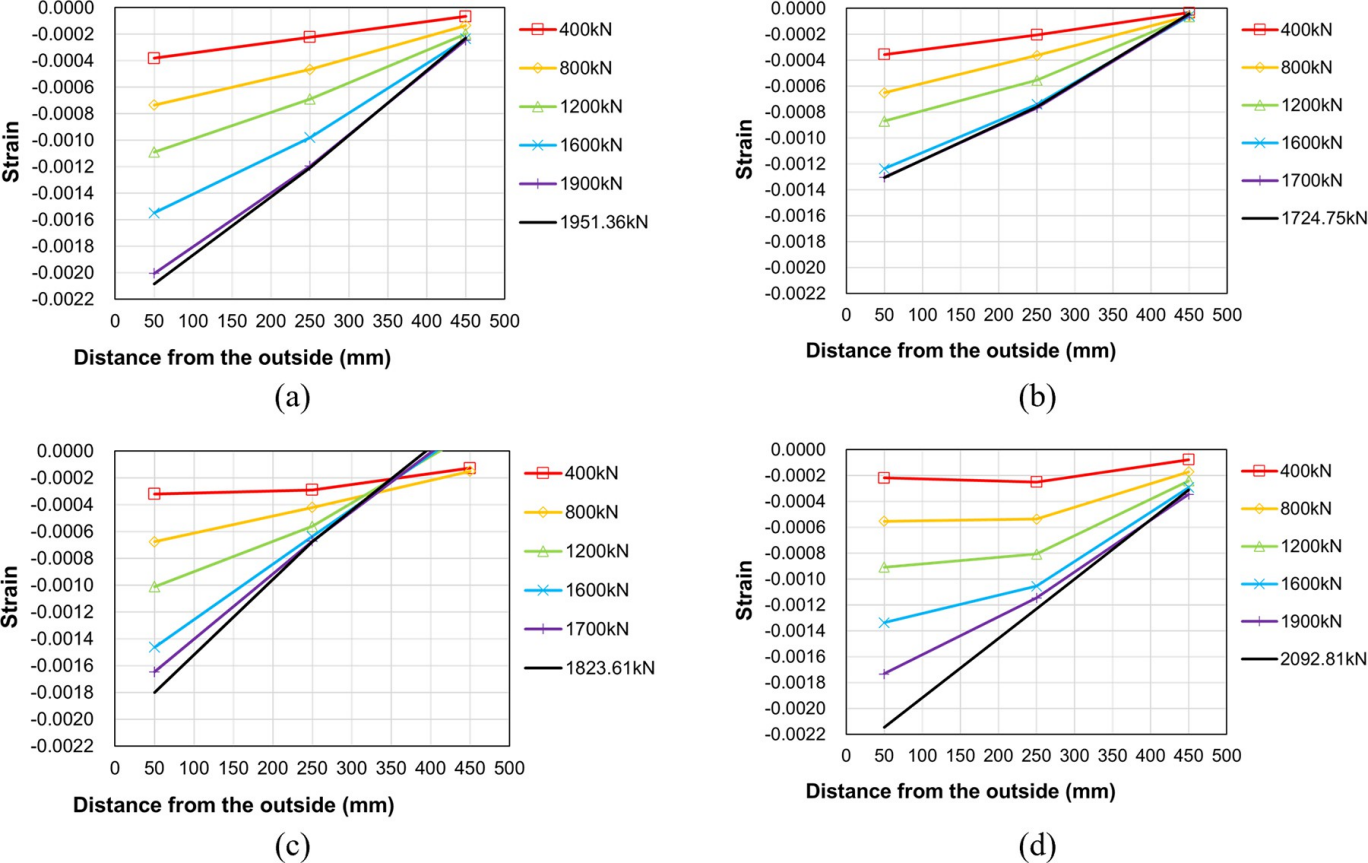
Concrete strain distribution near the end of the boundary beam (C4~C6). (a) Specimen B50-T125-S125. (b) Specimen B40-T125-S125. (c) Specimen B50-T100-S125. (d) Specimen B50-T125-S75.

**Fig 18:**
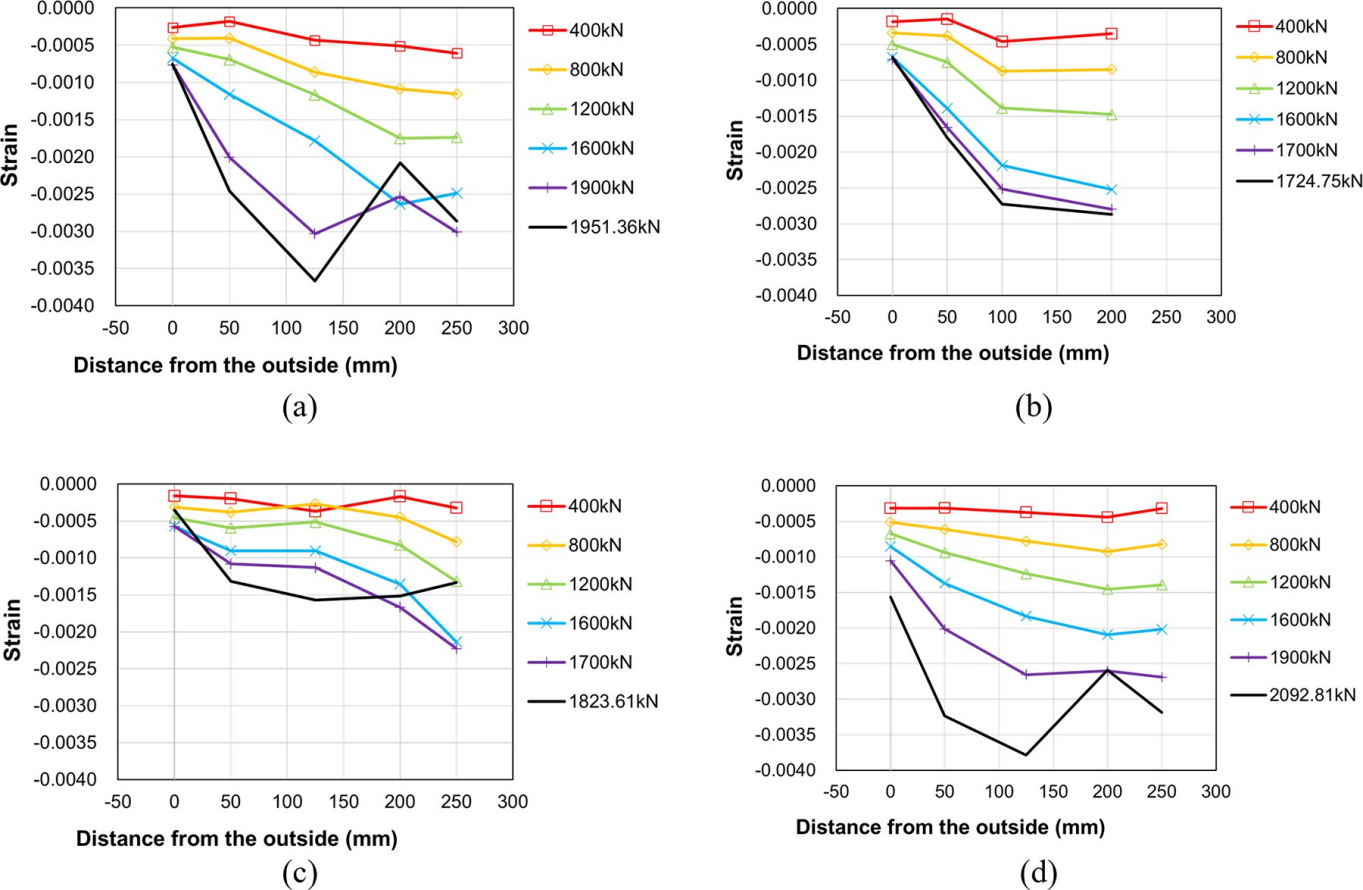
Concrete strain distribution at the lower wall (C10~C14). (a) Specimen B50-T125-S125. (b) Specimen B40-T125-S125. (c) Specimen B50-T100-S125. (d) Specimen B50-T125-S75.

To investigate the internal force transfer within the proposed boundary beam-wall system, the change in strain distribution with increasing load was analyzed for each specimen. Figs 16 to 18 plot the concrete strain distributions in the upper wall, the region near the end of the boundary beam and the lower wall, respectively. The horizontal axis denotes the strain gauge location, while the vertical axis represents the measured strain for several representative values of the applied load. It is crucial to emphasize that the strain gauges provide local rather than regional strain measurements. Consequently, it is noted that the data in close proximity to failure may not be entirely accurate. Fig 8 indicates the locations of the strain gauges used for data measurement.

Fig 16 presents the concrete strain measurement in the left upper wall. The strain magnitude diminishes as the distance from the outer surface increases, with the overall strain values significantly lower than the peak compressive strength strain, typically ranging between 0.002 and 0.003 for normal weight concrete [18, 19]. This suggests a lack of stress concentration within the upper wall. The results in the figure also reveal a decrease in some strain values at maximum load, likely due to internal stress redistribution. As failure approaches, the collapse of the lower wall appears to affect the strain distribution in the upper wall. The results in Fig 17 indicate that the concrete strain distribution near the left end of the boundary beam is similar to that of the upper wall, albeit with a greater magnitude overall than the values shown in Fig 16.

Fig 18 shows the concrete strain distribution in the left lower wall. It can be observed from the figure that the overall compressive strain values of two specimens B50-T125-S125 and B50-T125-S75 are higher than those of the other two specimens as they have relatively higher peak strengths than the other two. This indicates that the magnitude of concrete strain (or stress) of the lower wall is closely related to the peak strength of the entire boundary wall-beam system.

**Fig 19:**
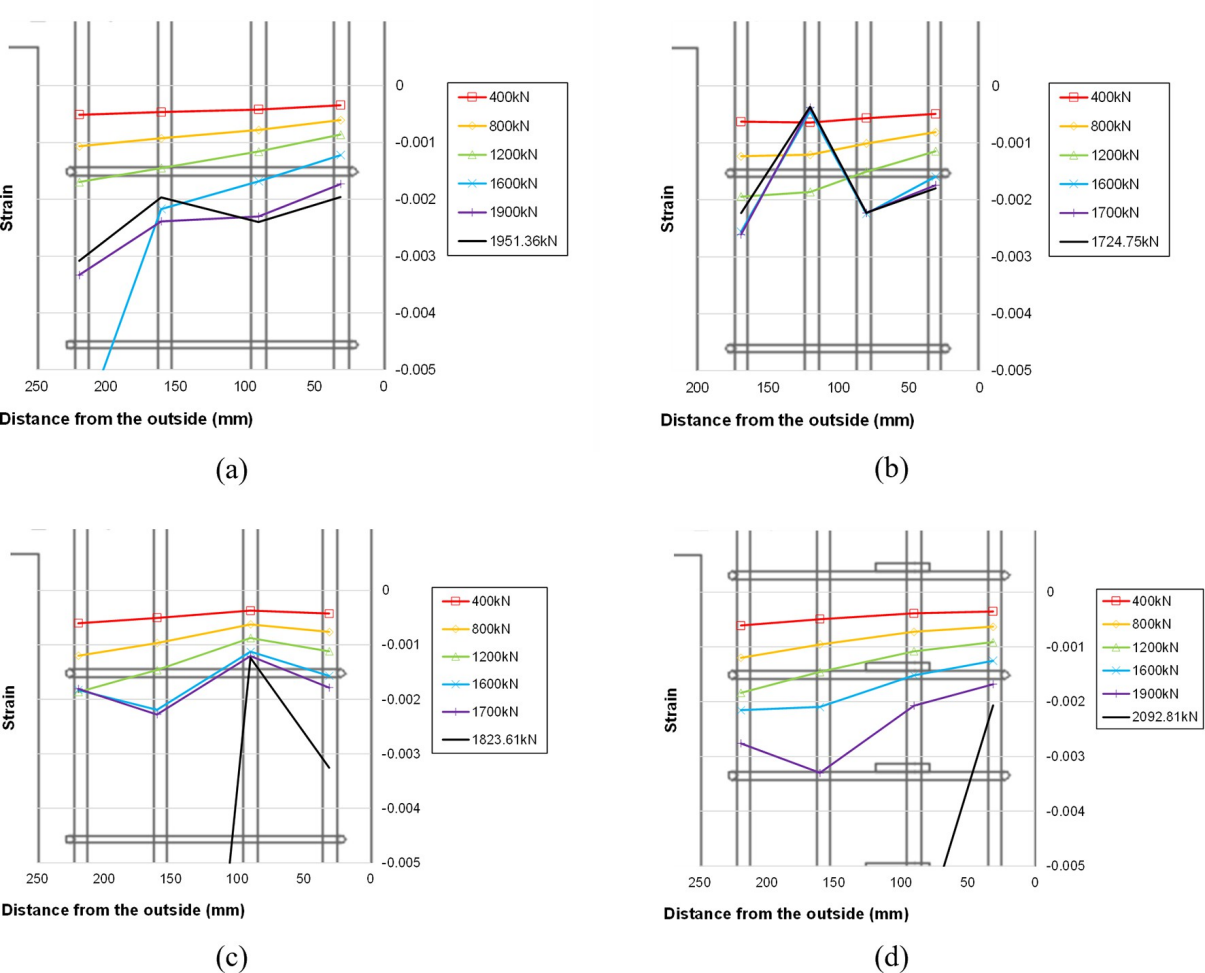
Main bar strain distribution at the top of the lower wall (S7~S10). (a) Specimen B50-T125-S125. (b) Specimen B40-T125-S125. (c) Specimen B50-T100-S125. (d) Specimen B50-T125-S75.

Fig 19 displays the strain distribution of the main bars in the right lower wall for each specimen. The horizontal axis represents the strain gauge location, while the vertical axis indicates the measured strain for several representative values of the applied load. It is evident from the Fig that specimen B50-T125-S75 exhibited a relatively uniform and gradual strain distribution of the main bars. In contrast, the other three specimens with 125mm stirrup spacing show a highly nonlinear strain distribution, suggesting the possibility of local buckling of the main bars in some regions at the peak strength.

**Fig 20:**
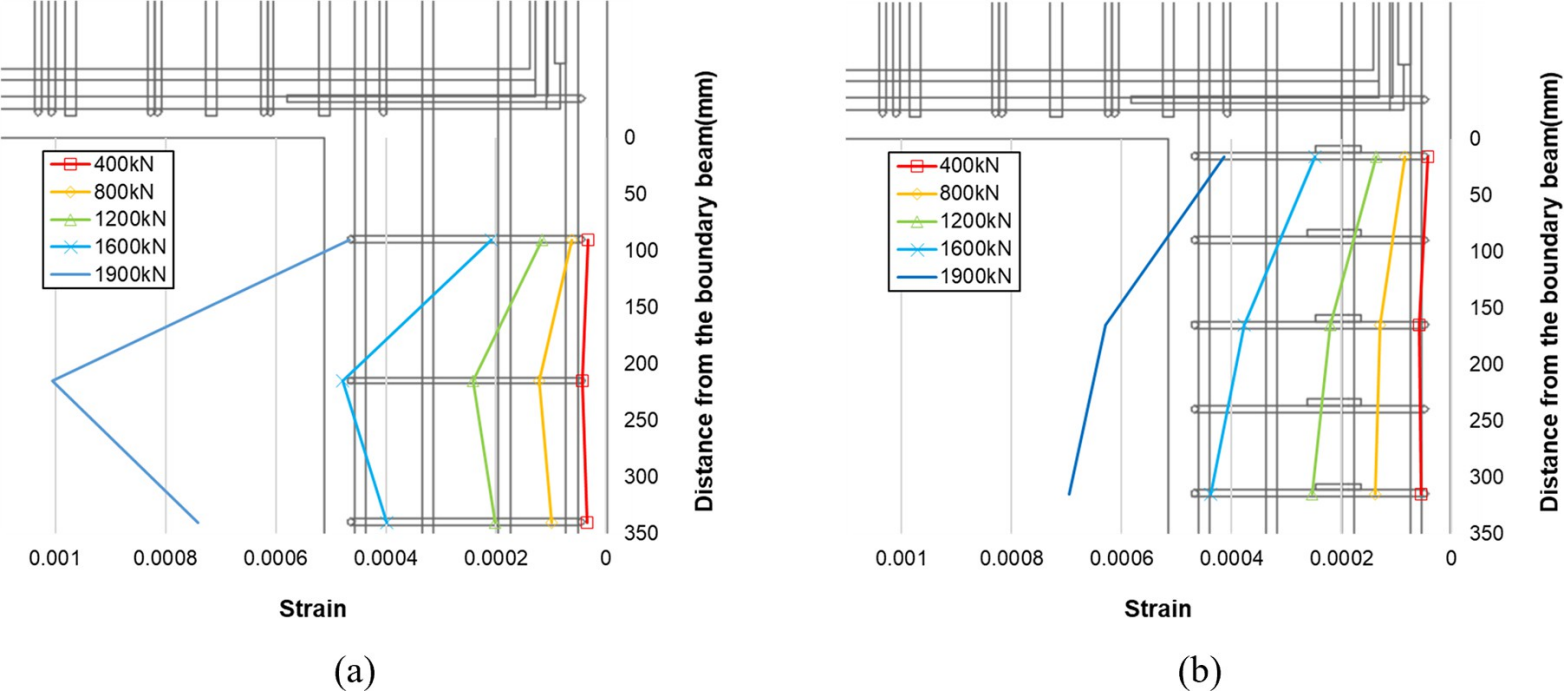
Stirrup strain distribution at the top of the lower wall (S11~S13). (a) Specimen B50-T125-S125. (b) Specimen B50-T125-S75.

Fig 20 depicts the strain distribution of the stirrup reinforcement in the right lower wall for two specimen B50-T125-S125 and B50-T125-S75. In this figure, the vertical axis represents the strain gauge location, while the horizontal axis indicates the measured strain for several representative values of the applied load. In the case of the reference specimen B50-T125-S125, a significant increase in strain is evident at the second stirrup below the boundary beam, and this trend becomes more pronounced with increasing load. In contrast, such behavior is not observed in the strain distribution of specimen B50-T125-S75, which is reinforced with stirrup reinforcement of smaller spacing and cross-ties. This confirms that the installation of stirrup reinforcement with smaller spacing and cross-ties is effective in alleviating local stress concentration in the lower wall, ultimately enhancing the maximum load-carrying capacity of the boundary beam-wall system.

**Fig 21:**
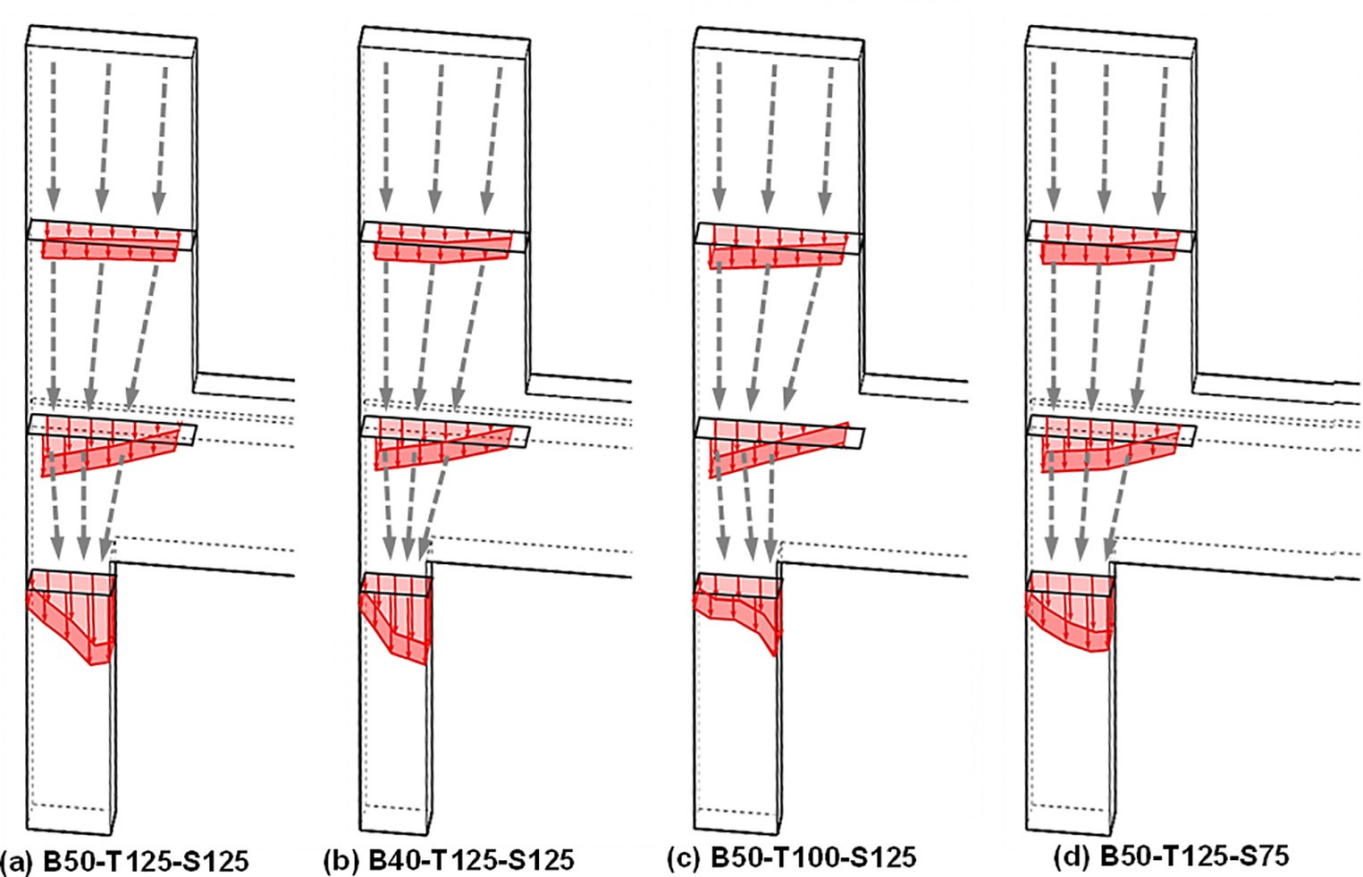
Load transfer path of each specimen.

Based on the preceding discussion, the load transfer path and concrete strain distribution at the three locations, where strain gauges were installed, are illustrated for each specimen in Fig 21. The concrete strain distributions presented in the figure represent the values measured under an applied load of 1,600 kN. By examining the concrete strain distributions depicted in the figure, the load transfer paths of each specimen are approximately illustrated. This analysis suggests that the effective stress region along the load transfer path diminishes as the cross-sectional area of the lower wall decreases. Additionally, enhanced implementation of shear reinforcement in the lower wall not only mitigates stress concentration but also facilitates the distribution of internal stress over a broader area.

## 4. Nonlinear finite element analysis

In this section, the outcomes of three-dimensional nonlinear finite element (FE) analysis conducted on the four test specimens are discussed. These results are then compared with those of the test to validate the efficacy of the proposed system. The analysis employed a widely recognized FE analysis software, Abaqus [20].

### 4.1. Finite element modeling

The compressive behavior of concrete in the nonlinear FE analysis relies on the concrete damage plasticity model introduced by Lee and Fenves [21], which enhances the concrete yield function proposed by Lubliner et al. [22]. The FE analysis employed the eccentricity ratio of 0.1 and stress ratio of 1.16, which are the default values in the concrete damage plasticity analysis of ABAQUS CAE software, as referenced in [20]. Additionally, the choice of the dilation angle of 35 degrees and *K*_*c*_ of 0.667 was made without accounting for viscosity by following the approaches introduced in [23, 24].

**Fig 22:**
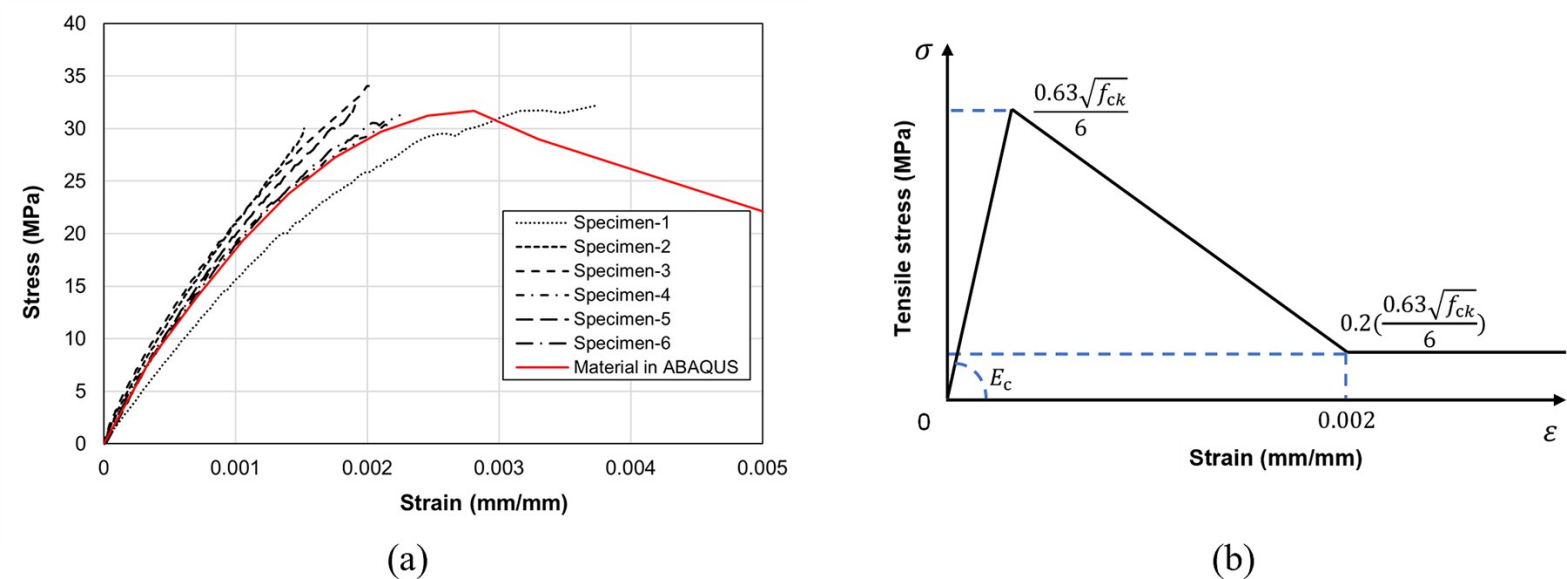
Stress-strain curves of concrete. (a) Compressive behavior. (b) Tensile behavior.

The compressive stress-strain relationship of concrete was mainly based on Hognestad model [25], and its shape was determined by combining the compression strength test results of the six cylindrical concrete specimens, as illustrated in Fig 22(A). The tensile stress-strain relationship of concrete was defined by the bilinear model illustrated in Fig 22(B), as proposed by Massicotte et al. [26]. Its peak strength was determined by multiplying the nominal tensile strength Eq (1) provided by ACI 318–19 [27] with a factor of 1/6.


fr=0.63fc', (1)

The material properties for concrete were derived from the compression strength tests conducted on cylindrical specimens, providing an elastic modulus of 22,574 MPa and a compressive strength of 31.7 MPa. To ensure convergence in the analysis, the curve is linearly decreased until reaching a strain value of 0.002, and thereafter, 20% of the peak tensile strength is maintained. The Poisson’s ratio used in the analysis was set at 0.167 by referring to [28–30]. For the stress-strain relationship of steel reinforcing bars, a perfect elasto-plastic model is employed, utilizing the von Mises yield criterion. The material properties of the D10 and D6 rebars listed in Table 3 were used in the FE analysis, along with a Poisson’s ratio of 0.3.

**Fig 23:**
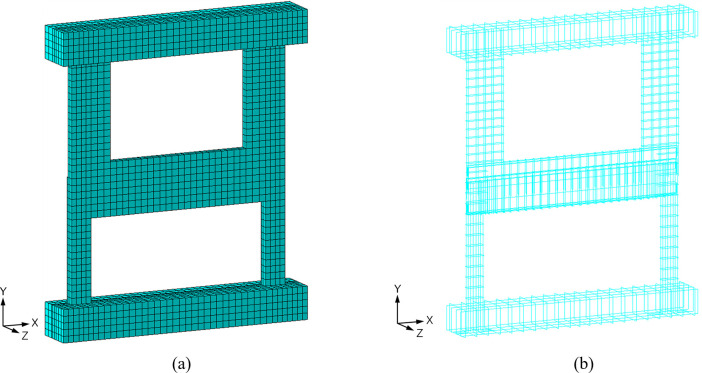
Finite element mesh adopted in this study. (a) Concrete. (b) Rebars.

Two types of elements were employed in the FE analysis: 20-node quadratic brick elements (C3D20) for the concrete portion and 3-node quadratic truss elements (T3D3) for the steel rebars. There elements are high-order elements, not linear ones, and are known for their excellent performance, particularly in plasticity analysis. The truss elements were integrated into the concrete region using the embedded region option provided by Abaqus. The mesh configuration of the FE model is depicted in Fig 23. The mesh size employed for the concrete frame and rebars is approximately 70 mm, roughly equivalent to the main bar spacing of the lower wall.

**Fig 24:**
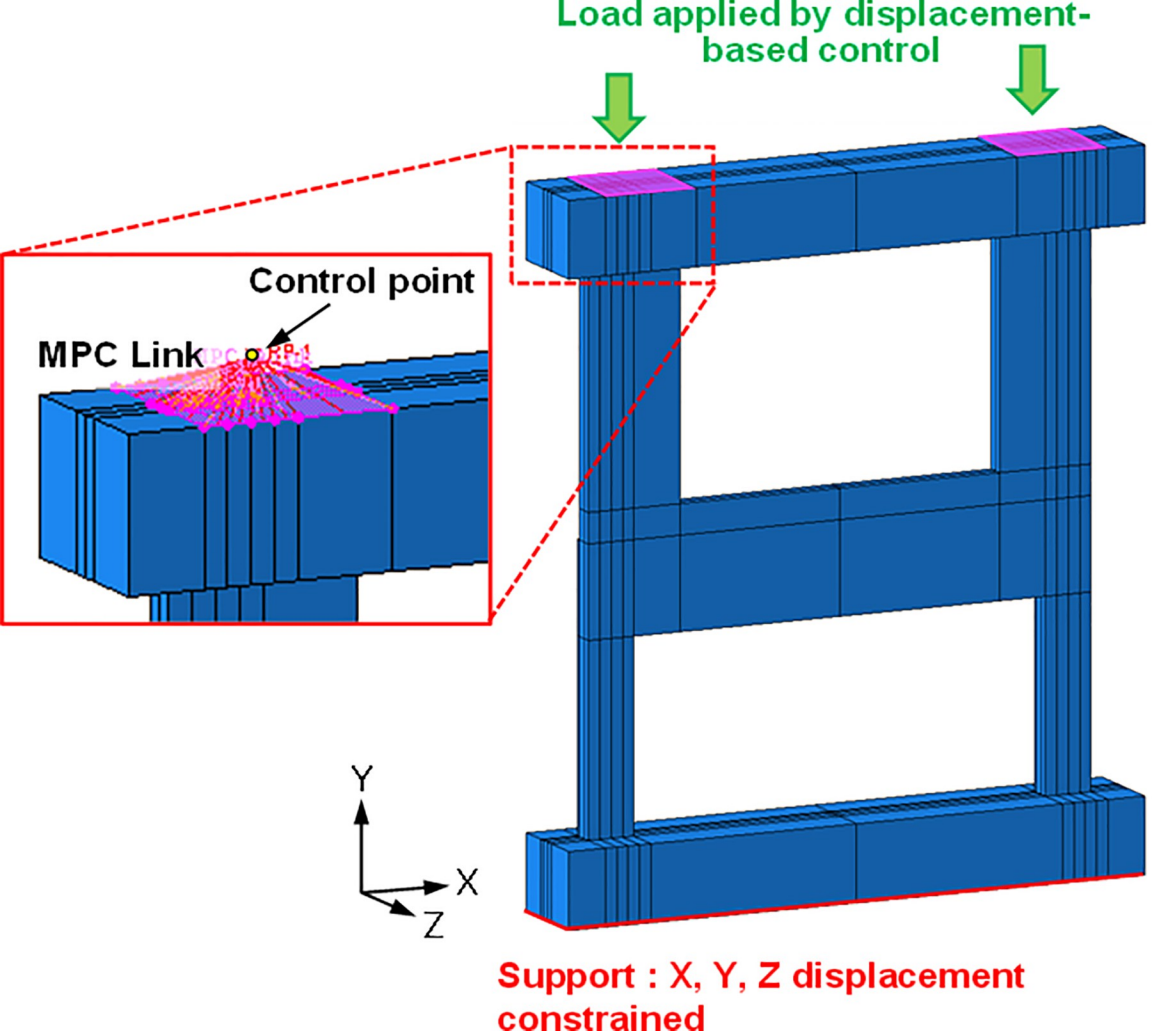
Boundary conditions of the finite element model.

The boundary conditions applied in the FE analysis are illustrated in Fig 24. All three *x*-, *y*-, and *z*- displacements were constrained at the bottom of the test specimen. As shown in the figure, a single control point was linked to multiple slave nodes on the control surface using multi-point constraint (MPC) links of rigid pin type [20]. This arrangement allows for the sharing of displacements, while preserving the distance between the control point and the constrained slave nodes. The load was applied in the form of displacement-based control by incrementally increasing the *y*-displacement at the two control points, while keeping their *z*-displacements constrained.

### 4.2. Comparison between the load-displacement curves by the test and FE analysis

**Fig 25:**
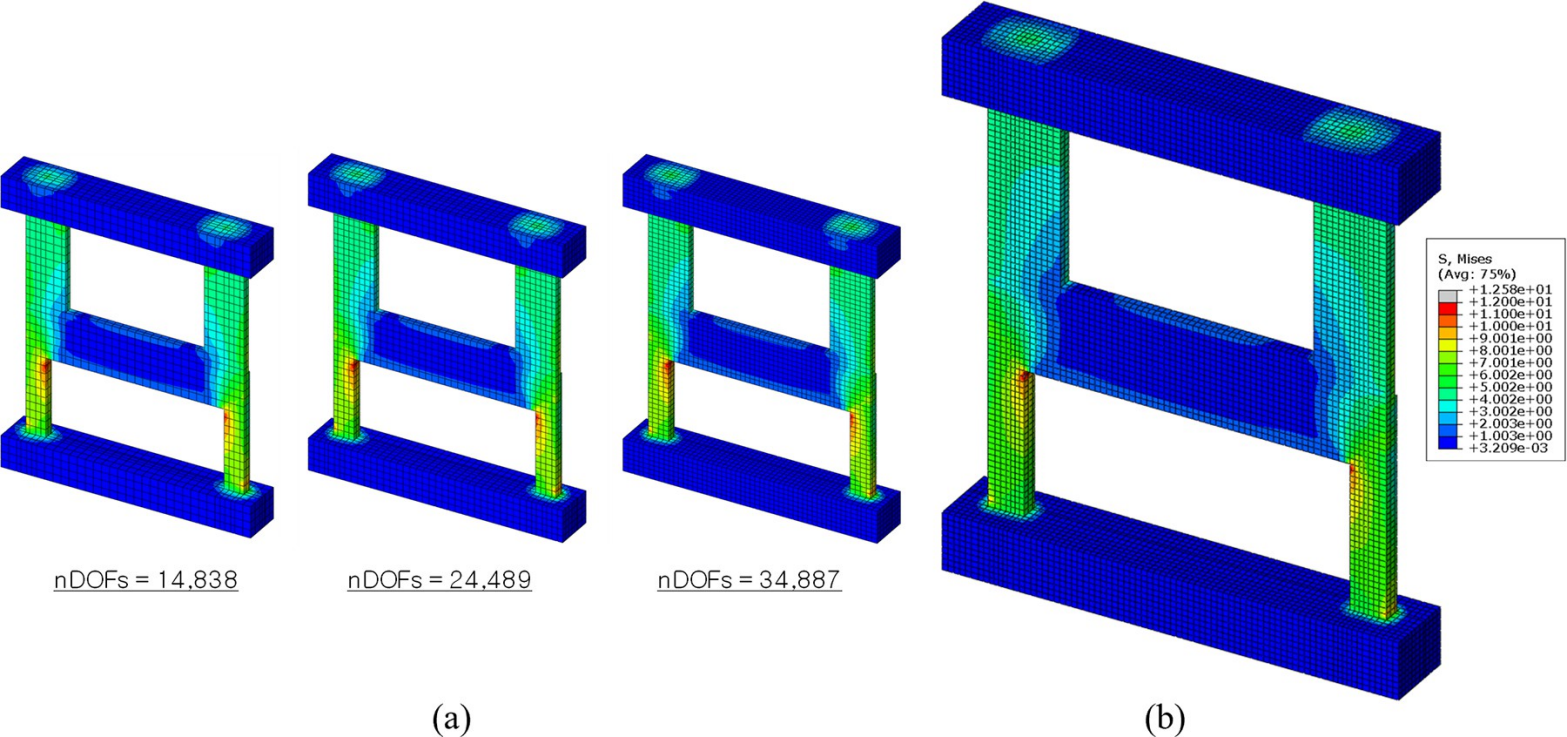
Finite element meshes used for sensitivity analysis. (a) Three different meshes used for sensitivity analysis. (b) Reference model.

A mesh sensitivity analysis was conducted to verify the effectiveness of the finite element mesh used in this study, as depicted in Fig 23. Fig 25(A) presents the three meshes employed for this analysis. The numbers of degrees of freedom (nDOFs) for these meshes are 14,838, 24,489, and 34,887, respectively. The finite element mesh displayed in Fig 23 matches the one with 24,489 nDOFs among the three models shown in Fig 25(A).

To evaluate the accuracy of their solutions, the displacements (*d*_*i*_) at the locations of LVDT-2 and 3 were measured under a load of 500 kN. These displacements are compared with those of the reference model, illustrated in Fig 25(B), by calculating the relative error in displacement (*e*_*r*_) as follows:

$$e_{r}=|\frac{d_{i}-d_{ref}}{d_{ref}}|,$$ (2)

where *d*_*i*_ and *d*_*ref*_ represent the measured displacements for each of the three models and the reference model, respectively. The reference model features a highly refined mesh, as depicted in Fig 25(B), with nDOFs of 95,307, significantly higher than those of the three models shown in Fig 25(A). Although this model yields highly accurate solutions, it requires a substantially longer analysis time compared to the other three models.

**Fig 26:**
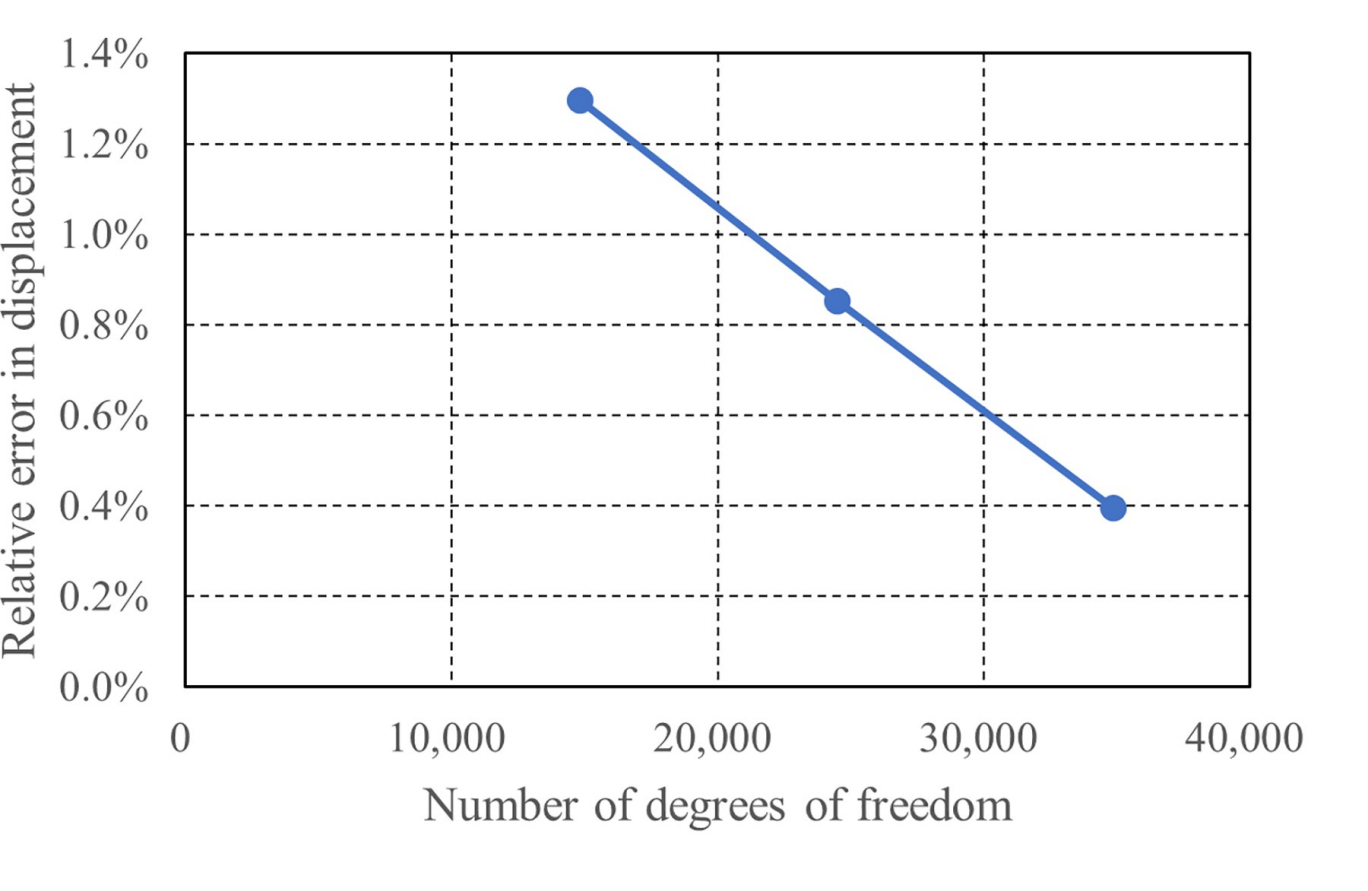
Relative errors in displacement of the three models with respect to nDOFs.

The results of the mesh sensitivity analysis are summarized in Table 5, which includes the nDOFs, measured displacements and the relative errors in displacement for the three models and the reference model. The relative errors in displacement for the three models are plotted against their nDOFs in Fig 26. Both the table and the figure demonstrate that the model with 24,489 nDOFs, which corresponds to the mesh shown in Fig 23, delivers adequately accurate solutions with a relative error of less than 1%. This finding supports the selection of this particular mesh for the detailed numerical investigation of the test results.

**Table 5:** Results of the mesh sensitivity analysis.

Model ID	nDOFs	Measured displacement (mm)	Relative error in displacement (%)
1	14,838	0.77681	1.296
2	24,489	0.78031	0.851
3	34,887	0.78390	0.395
Reference	95,307	0.78701	0.000

**Fig 27:**
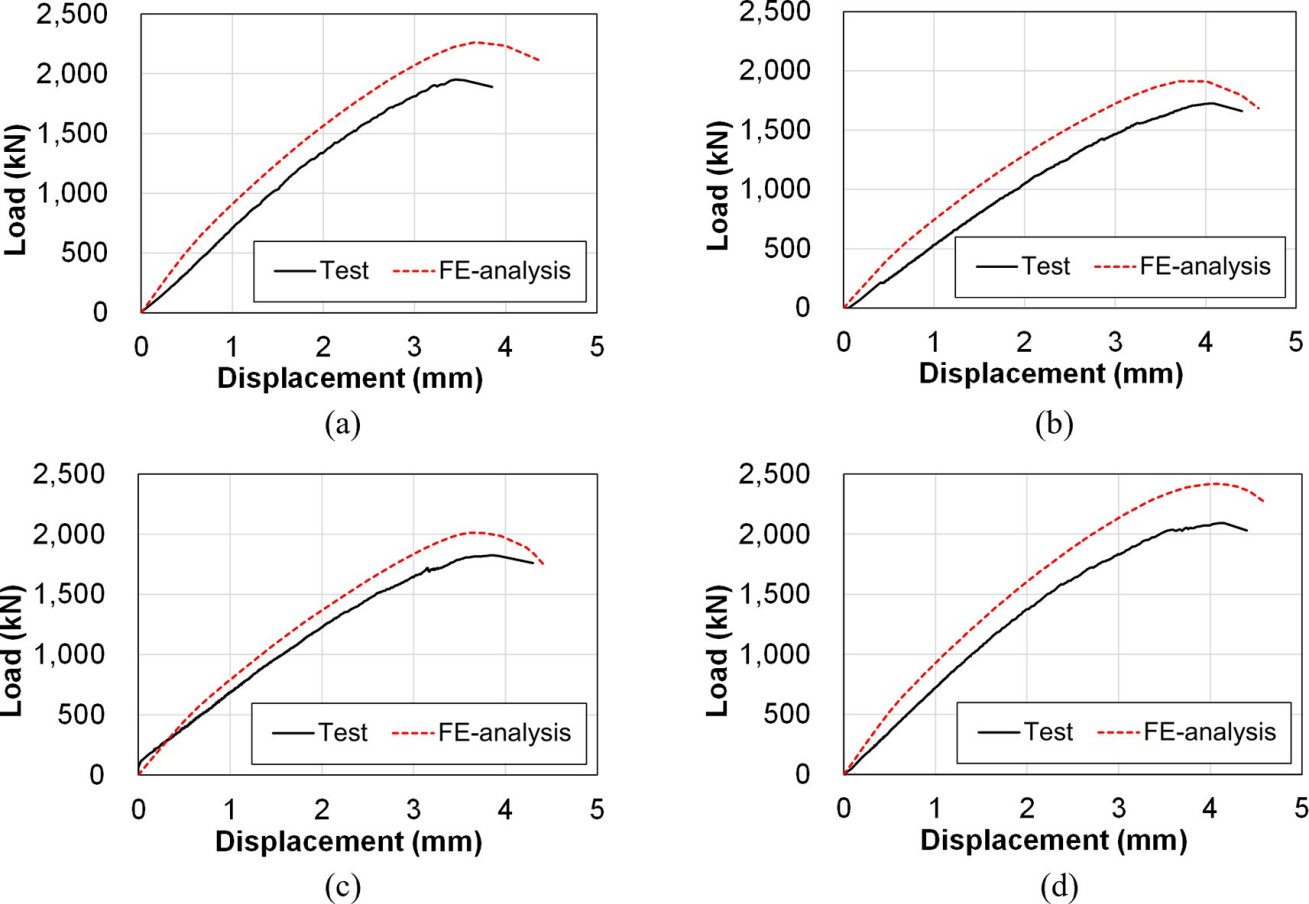
Comparison between the load-displacement curves by the test and FE analysis. (a) Specimen B50-T125-S125. (b) Specimen B40-T125-S125. (c) Specimen B50-T100-S125. (d) Specimen B50-T125-S75.

The load-displacement curves of the four specimens obtained from the test and FE analysis results are plotted in Fig 27. Displacement was measured at the location of LVDT-2 and 3, as similar to the case of Fig 15. The values of peak strength and tangent modulus for each specimen, obtained from both the test and FE analysis, are summarized in Table 6. The relative error of the FE analysis values compared to the test values is also provided in the table. The tangent modulus value is defined as the slope between displacements of 0.8 mm to 1.5 mm, excluding the initial portion where the slopes of the two load-displacement curves do not coincide with each other.

**Table 6:** Initial stiffness and peak strength of each specimen by the test and FE analysis.

Specimen	Peak strength			Tangent modulus		
	FE analysis (kN)	Test (kN)	Relative error (%)	FE analysis (kN/mm)	Test (kN/mm)	Relative error (%)
B50-T125-S125	2,263.61	1,951.36	16.00	702.86	685.71	2.50
B40-T125-S125	1,911.87	1,724.75	10.85	585.71	585.71	6.49
B50-T100-S125	2,012.34	1,823.61	10.35	614.29	581.43	5.65
B50-T125-S75	2,417.58	2,092.81	15.52	725.71	690.00	5.18

It is evident from the table that the peak strengths obtained from the FE analysis are approximately 10 to 16% higher than those from the test results. This discrepancy can be primarily attributed to the eccentricity of the applied load and manufacturing variations in the test specimens. These factors exert a more significant impact in compression tests compared to other types, such as bending or cyclic lateral loading tests. The relative error in the tangent modulus ranges only from 2.5 to 6.5%, indicating that the structural behavior of the specimen obtained from the FE analysis is very similar to that of the test.

Table 7 summarizes the peak strength values obtained from the FE analysis and the test for each specimen. It also presents the relative values of the peak strength, estimated with respect to the reference specimen (B50-T125-S125). It can be observed from the table that the relative ratios of the FE analysis and test generally align well with each other. This demonstrates a consistent trend between the FE analysis and test results, indicating that the FE model developed in this study can accurately predict the structural behavior of the test specimens.

**Table 7:** Relative ratio of the peak strength of each specimen to that of the reference specimen.

Specimen	FE analysis		Test	
	Peak strength (kN)	Relative ratio (%)	Peak strength (kN)	Relative ratio (%)
B50-T125-S125	2,263.61	100.0	1,951.36	100.0
B40-T125-S125	1,911.87	84.5	1,724.75	88.4
B50-T100-S125	2,012.34	88.9	1,823.61	93.5
B50-T125-S75	2,417.58	106.8	2,092.81	107.2

### 4.3. Stress distribution by FE analysis

**Fig 28:**
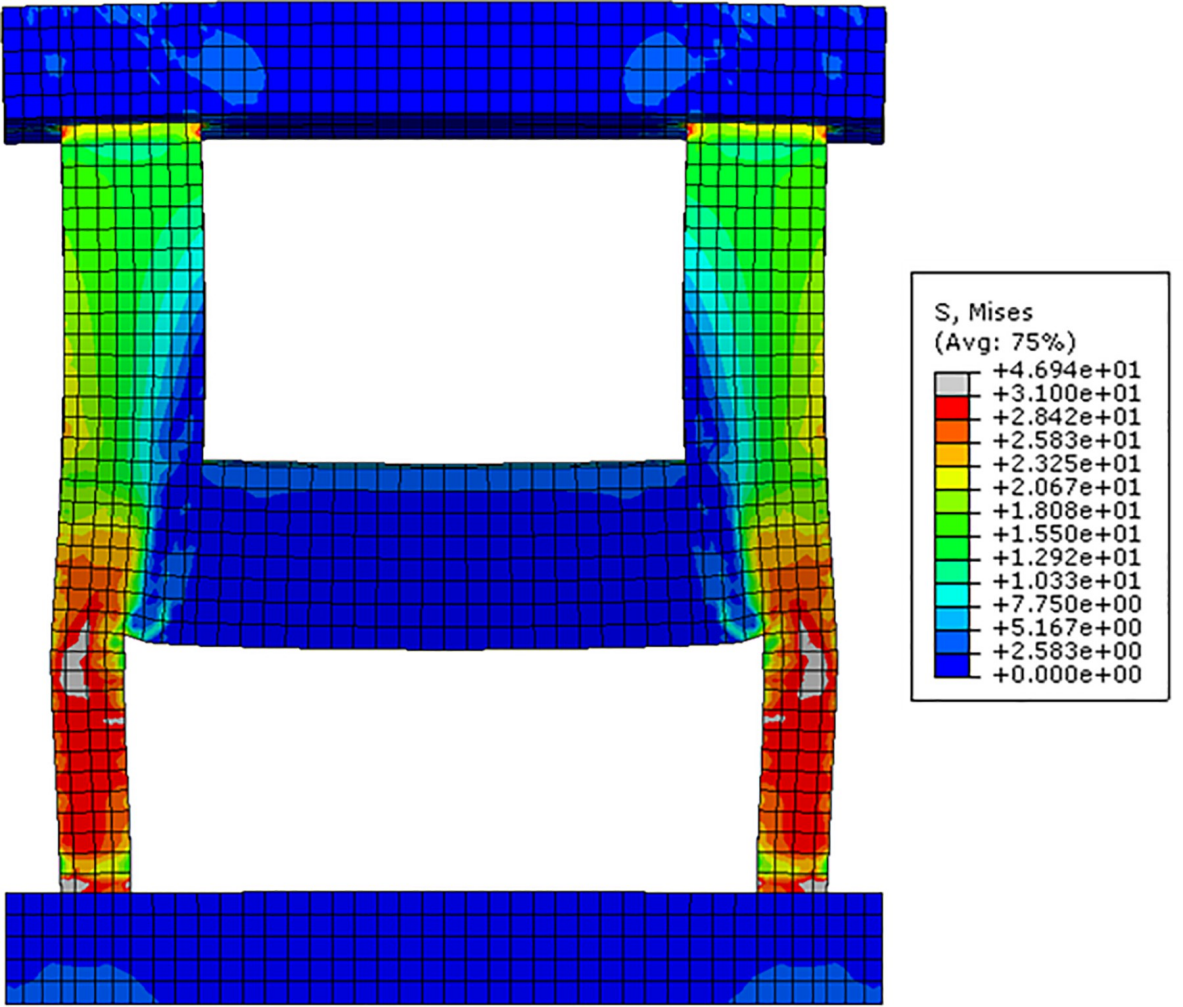
Von Mises stress distribution at peak load (Reference specimen B50-T125-S125, stress unit: MPa).

The typical deformed shape and von Mises stress distribution of the test specimen at its peak strength are shown in Fig 28. These results were obtained from the FE analysis of the reference specimen B50-T125-S125, with a scale factor of 30.0 applied to enhance the clarity of the deformed shape. As shown in the figure, the lower walls, experiencing compression forces, exhibit an outward convex bending, significantly contributing to the overall failure of the test specimen. This closely resembles the actual failure mode of the test specimen, as observed in Fig 8. Additionally, there is evident stress concentration in the outwardly curved lower wall region, which coincides well with the strain amplification phenomenon observed in the stirrup reinforcement at the same location, as can be identified in Fig 20(A).

**Fig 29:**
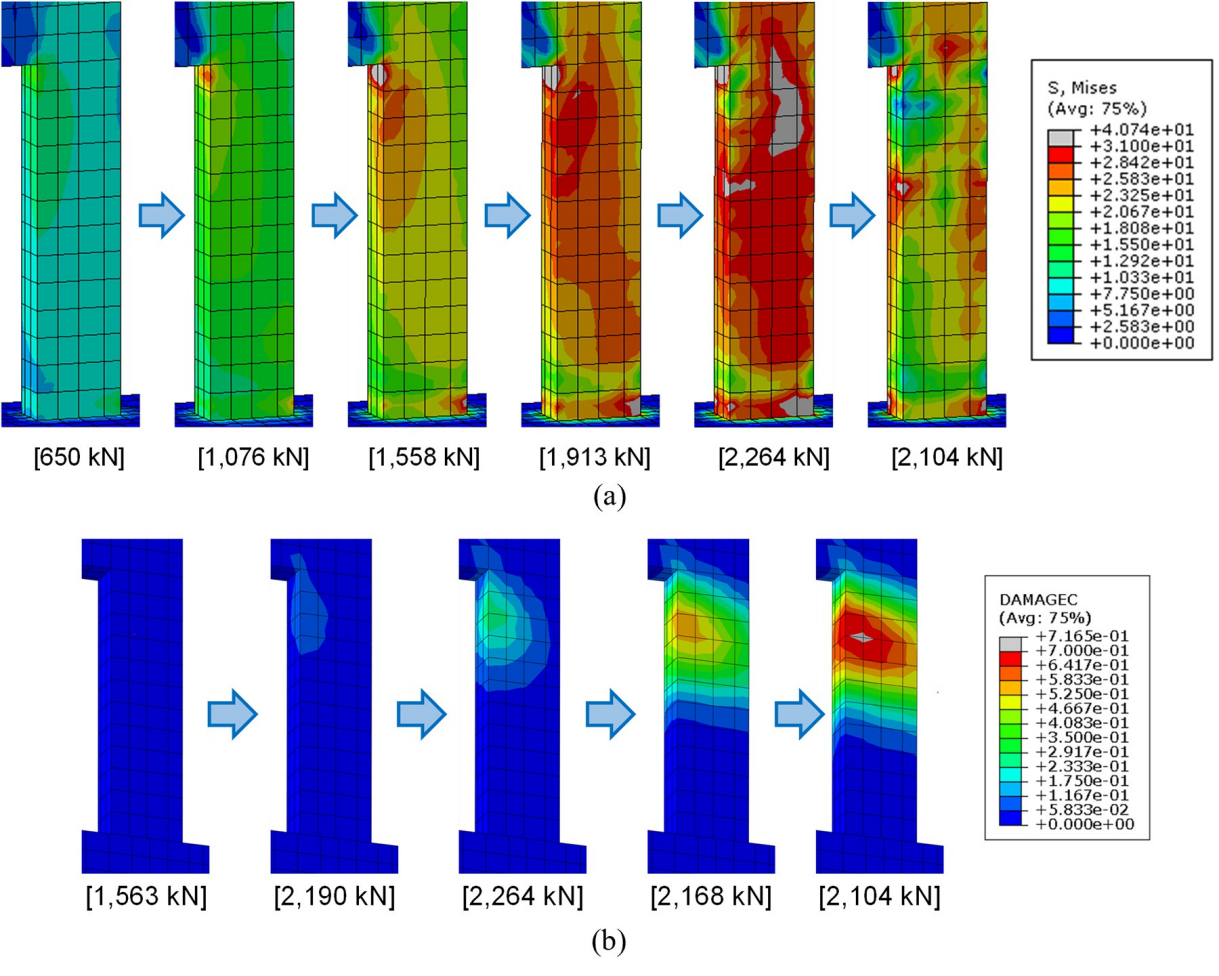
Von Mises stress and DAMAGEC distributions of the lower wall concrete at several different loading stages (Reference specimen B50-T125-S125). (a) Von Mises stress distribution. (Stress unit: MPa). (b) DAMAGEC distribution.

**Fig 30:**
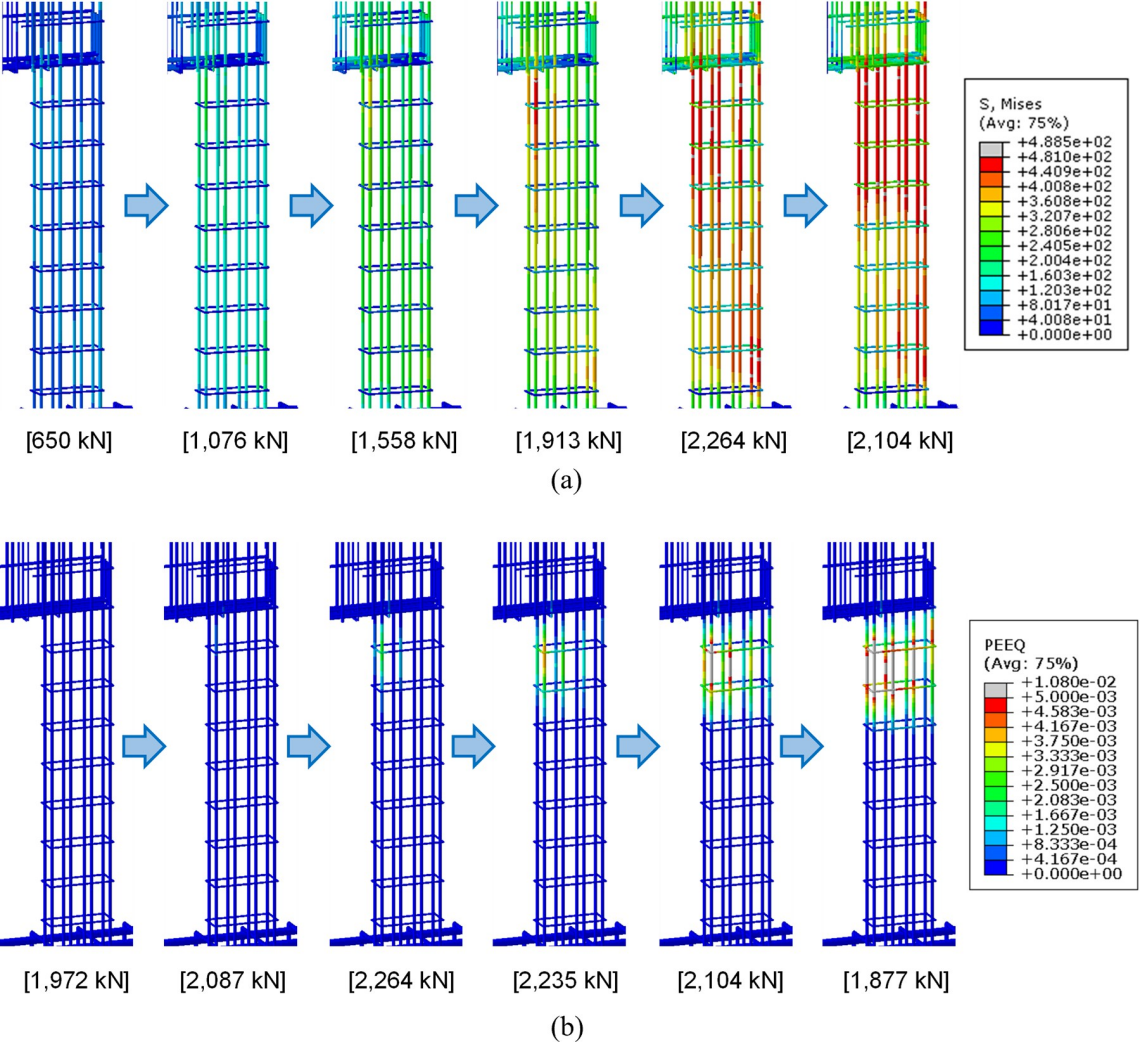
Von Mises stress and plastic strain distributions of the lower wall rebars at several different loading stages (Reference specimen B50-T125-S125). (a) Von Mises stress distribution. (Stress unit: MPa). (b) Plastic strain distribution.

Von Mises stress and compressive damage (DAMAGEC) distributions of the lower wall concrete are presented at several different values of the applied load (kN) in Fig 29. Similarly, von Mises stress and plastic strain distributions of the lower rebars are also displayed in Fig 30. These results are obtained from the FE analysis of the reference specimen B50-T125-S125. These distributions offer valuable insights into the progression of the test specimen failure. The results in Fig 29(A) reveal that as the load increases, stress gradually concentrates in the outwardly curved lower wall region depicted in Fig 28. Most of this region experiences yielding at the peak strength of 2,264 kN. Subsequently, as the applied load is reduced to 2,104 kN, von Mises stress in this region decreases, which is evident from the final stress distribution of Fig 29(A). However, during this process, von Mises stress and plastic strain in the rebars within the same region are intensified, as shown in Figs 30(A) and 30(B). Similarly, compressive damage in the lower concrete intensifies even after the peak load is surpassed, as shown in Fig 29(B). This pattern suggests a significant redistribution of internal forces from the concrete to the rebars in the specified region, occurring as the concrete reaches its yield strength and becomes incapable of sustaining further internal forces.

**Fig 31:**
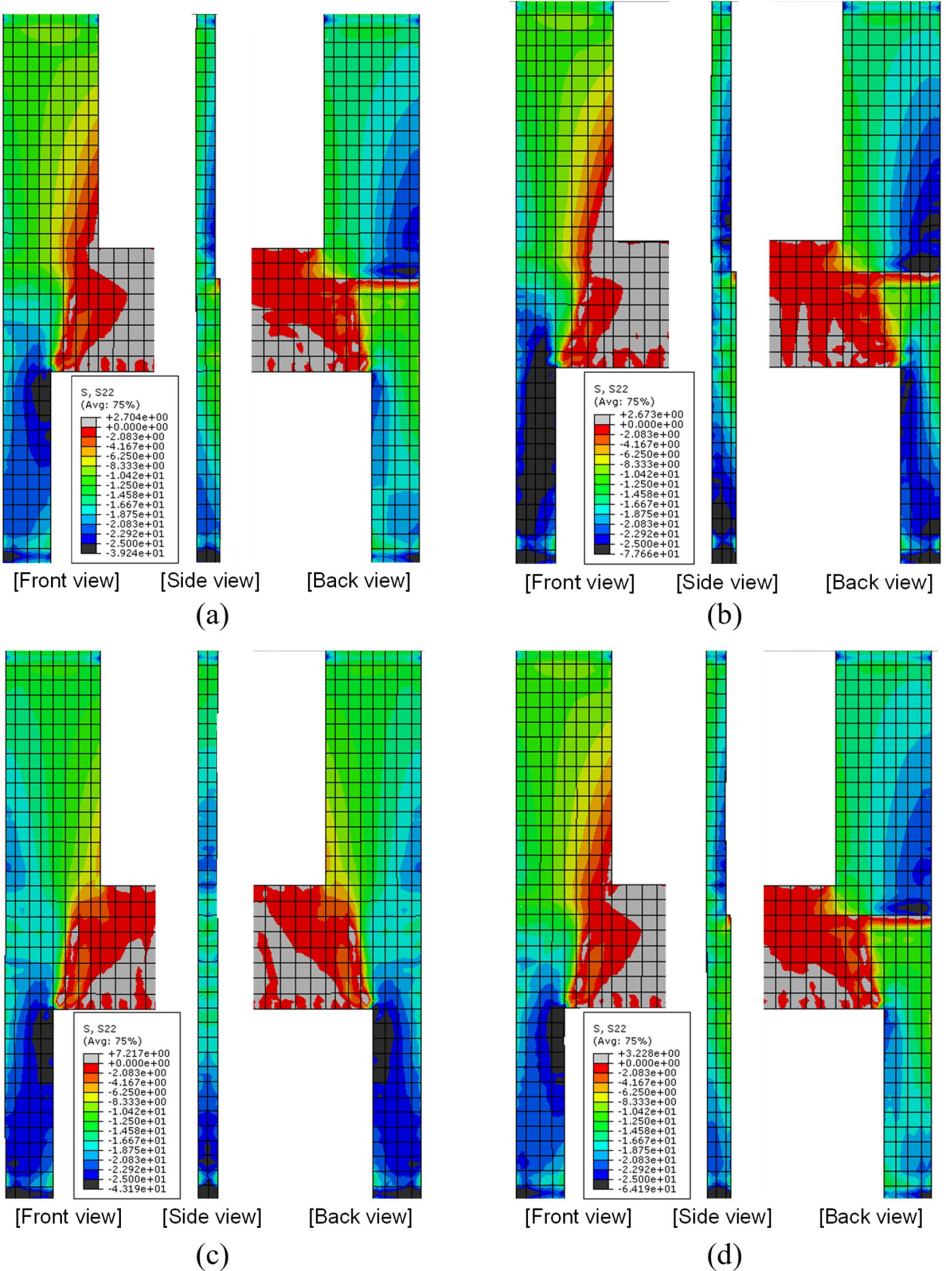
S22 stress distribution near the top of the lower wall. (Stress unit: MPa). (a) Specimen B50-T125-S125. (b) Specimen B40-T125-S125. (c) Specimen B50-T100-S125. (d) Specimen B50-T125-S75.

To elucidate the load transfer path, the normal stress distribution of concrete in the vertical direction obtained from the FE analysis are shown for each specimen in Fig 31. These stress distributions are obtained from the FE analysis results at the applied load of 1,600 kN, as similar to the case shown in Fig 21. The results in the figure reveal that, in specimen B50-T100-S125, the stress distributions on the front and back sides coincide well with each other, whereas they do not in all other specimens. This discrepancy can be primarily attributed to the fact that specimen B50-T100-S125 features identical thicknesses for the upper and lower walls, while the others have a lower wall thickness greater than that of the upper wall. In the latter case, stress concentration occurs at the boundary of the upper and lower walls due to induced bending moments in the out-of-plane direction caused by the differing thicknesses. Consequently, uneven stress distribution occurs in the upper wall, which is a phenomenon absent in the result of specimen B50-T100-S125.

From these observations, it can be inferred that the difference in upper and lower wall thicknesses results in uneven stress concentration within the boundary beam-wall system, leading to a compromised structural performance in terms of initial stiffness and peak strength, as discussed in Section 3.1. However, in practical wall-type apartment structures, it is anticipated that slabs function as diaphragms and contribute some lateral stiffness to the wall, as shown in Fig 2. This may mitigate the adverse effects of uneven upper and lower thicknesses.

## 5. Conclusions

In this study, the structural performance of the newly proposed boundary beam-wall system subjected to axial compression was investigated by performing a structural test on four specimens of 1/2 scale. In addition, three-dimensional nonlinear finite element analysis was also performed to verify the effectiveness of the proposed system. Key test parameters included the ratio of lower to upper wall lengths, the thickness of the lower wall, and the specifics of the stirrup reinforcement in the lower wall. For each specimen, a load-displacement curve was plotted, and the corresponding failure mode was identified. The main conclusions of this paper are as follows:

1. All the test specimens did not exhibit significant cracks or deformation until the applied vertical load reached its peak value. At the point of peak load, concrete crushing occurred on the upper inner side of the lower wall, and concurrently, the main bars of the lower wall buckled, eventually resulting in a rapid and catastrophic failure.
2. There was evident stress concentration in the outwardly curved lower wall region of the test specimen, which coincides well with the strain amplification phenomenon observed in the stirrup reinforcement at the same location.
3. The decrease in the lower wall length-to-upper wall length ratio significantly reduced the peak strength of the boundary beam-wall system, but its impact on the initial stiffness was less significant.
4. The difference in upper and lower wall thicknesses resulted in lateral bending caused by eccentricity in the out-of-plane direction and uneven stress concentration within the boundary beam-wall system, leading to a compromised structural performance in terms of initial stiffness and peak strength.
5. The use of cross-ties and a smaller stirrup distance in the lower wall significantly enhanced the initial stiffness and peak strength of the boundary beam-wall system, ultimately reducing stress concentration.
6. The effective stress region along the load transfer path diminished as the cross-sectional area of the lower wall decreased. Additionally, enhanced implementation of shear reinforcement in the lower wall facilitated the distribution of internal stress over a broader area.
7. The peak strengths obtained from the FE analysis were approximately 10 to 16% higher than those from the test results due to the eccentricity of the applied load and manufacturing variations in the test specimens. The relative error of the FE tangent modulus value to that of the test ranged only from 2.5 to 6.5%, indicating that the structural behavior of the specimen obtained from the FE analysis is very similar to that of the test.

Currently, the structural performance of the boundary beam-wall system under lateral cyclic loading is being investigated. This involves conducting tests on four 1/2-scale specimens, similar to those studied in this research, while simultaneously developing a three-dimensional nonlinear finite element model specifically for these specimens.

## Supporting information

S1 Data (XLSX)
